# eHealth-Based Psychosocial Interventions for Adults With Insomnia: Systematic Review and Meta-analysis of Randomized Controlled Trials

**DOI:** 10.2196/39250

**Published:** 2023-03-14

**Authors:** Wenrui Deng, Rianne M J J van der Kleij, Hongxia Shen, Junjie Wei, Evelyn A Brakema, Nick Guldemond, Xiaoyue Song, Xiaoming Li, Marie-José van Tol, André Aleman, Niels H Chavannes

**Affiliations:** 1 Department of Public Health and Primary Care Leiden University Medical Center Leiden Netherlands; 2 Department of Medical Psychology School of Mental Health and Psychological Science Anhui Medical University Hefei China; 3 Cognitive Neuroscience Center Department of Biomedical Sciences of Cells and Systems University Medical Center Groningen Groningen Netherlands; 4 School of Nursing Guangzhou Medical University Guangzhou China

**Keywords:** eHealth, psychosocial interventions, insomnia, adults, meta-analysis, mobile phone

## Abstract

**Background:**

Worldwide, insomnia remains a highly prevalent public health problem. eHealth presents a novel opportunity to deliver effective, accessible, and affordable insomnia treatments on a population-wide scale. However, there is no quantitative integration of evidence regarding the effectiveness of eHealth-based psychosocial interventions on insomnia.

**Objective:**

We aimed to evaluate the effectiveness of eHealth-based psychosocial interventions for insomnia and investigate the influence of specific study characteristics and intervention features on these effects.

**Methods:**

We searched PubMed, Embase, Web of Science, PsycINFO, and the Cochrane Central Register of Controlled Trials from database inception to February 16, 2021, for publications investigating eHealth-based psychosocial interventions targeting insomnia and updated the search of PubMed to December 6, 2021. We also screened gray literature for unpublished data. Eligible studies were randomized controlled trials of eHealth-based psychosocial interventions targeting adults with insomnia. Random-effects meta-analysis models were used to assess primary and secondary outcomes. Primary outcomes were insomnia severity and sleep quality. Meta-analyses were performed by pooling the effects of eHealth-based psychosocial interventions on insomnia compared with inactive and in-person conditions. We performed subgroup analyses and metaregressions to explore specific factors that affected the effectiveness. Secondary outcomes included sleep diary parameters and mental health–related outcomes.

**Results:**

Of the 19,980 identified records, 37 randomized controlled trials (13,227 participants) were included. eHealth-based psychosocial interventions significantly reduced insomnia severity (Hedges *g*=−1.01, 95% CI −1.12 to −0.89; *P*<.001) and improved sleep quality (Hedges *g*=−0.58, 95% CI −0.75 to −0.41; *P*<.001) compared with inactive control conditions, with no evidence of publication bias. We found no significant difference compared with in-person treatment in alleviating insomnia severity (Hedges *g*=0.41, 95% CI −0.02 to 0.85; *P*=.06) and a significant advantage for in-person treatment in enhancing sleep quality (Hedges *g*=0.56, 95% CI 0.24-0.88; *P*<.001). eHealth-based psychosocial interventions had significantly larger effects (*P*=.01) on alleviating insomnia severity in clinical samples than in subclinical samples. eHealth-based psychosocial interventions that incorporated guidance from trained therapists had a significantly greater effect on insomnia severity (*P*=.05) and sleep quality (*P*=.02) than those with guidance from animated therapists or no guidance. Higher baseline insomnia severity and longer intervention duration were associated with a larger reduction in insomnia severity (*P*=.004). eHealth-based psychosocial interventions significantly improved each secondary outcome.

**Conclusions:**

eHealth interventions for insomnia are effective in improving sleep and mental health and can be considered a promising treatment for insomnia. Our findings support the wider dissemination of eHealth interventions and their further promotion in a stepped-care model. Offering blended care could improve treatment effectiveness. Future research needs to elucidate which specific intervention components are most important to achieve intervention effectiveness. Blended eHealth interventions may be tailored to benefit people with low socioeconomic status, limited access to health care, or lack of eHealth literacy.

## Introduction

### Background

Insomnia is a common complaint in primary care and a prevalent public health problem [1]. The Diagnostic and Statistical Manual of Mental Disorders, Fifth Edition, defines insomnia as dissatisfaction with sleep quantity or quality, characterized by difficulty initiating or maintaining sleep for 3 or more days per week for a minimum duration of 3 months, accompanied by considerable distress and functional impairments (eg, intellectual-, behavioral-, social-, occupational-, and mood-related impairment) [2]. Approximately 25% of adults experience unsatisfactory sleep, and approximately 6% to 10% of adults meet the diagnostic criteria for insomnia [1]. In low-income settings in Africa and Asia, the prevalence of sleep problems can reach up to 40% [3]. Insomnia is often persistent and debilitating, increasing the risk of other physical or mental illnesses or exacerbating existing medical or psychiatric disorders [4,5]. Thus, insomnia carries a heavy individual and societal burden, including the burden on the health care system, which hinders societal development and leads to socioeconomic losses [6,7].

Although a range of pharmacologic treatments and psychosocial therapies exist for insomnia, the treatment of insomnia remains a major challenge [8]. Previous research has demonstrated that benzodiazepines and benzodiazepine receptor agonists have a short-term efficacy on insomnia, whereas long-term use is usually associated with potential side effects, including memory dysfunction, somatic symptoms, drug dependence, and interactions [9]. Cognitive behavioral therapy (CBT) for insomnia is effective with long-lasting effects when compared with medications, and is recommended as the first-line treatment for insomnia [10]. However, because of the limited number of trained therapists, high costs, and time-intensive nature of in-person CBT for insomnia (CBT-I), millions of patients still do not have access to this effective treatment to improve their sleep outcomes [11]. Hence, there is an urgent need for inexpensive, innovative delivery modalities of CBT or novel treatment options to be effective and accessible for the larger population at a lower cost [12,13].

eHealth is increasingly being developed and implemented for the delivery of remote, timely, high-quality, and limited-contact care [14]. eHealth can be defined as “health services and information delivered or enhanced through the Internet and related technologies” [15]. In a broader sense, it can encompass a range of services or systems that facilitate health care practice through the use of information and communication technologies, including electronic health records, e-prescriptions, digital interventions, telemedicine, and mobile health [16]. Furthermore, eHealth is increasingly being applied to the prevention and treatment of several mental illnesses, including but not limited to smoking cessation, anxiety, depression, and suicidal ideation [17-20]. In these eHealth programs, information about illness, treatment, self-management strategies, health status tracking, support, and feedback are delivered via the internet and related technologies [21-24]. Their results consistently show eHealth to have high accessibility, interactivity, and effectiveness with limited cost. In addition, a meta-analysis revealed the effects of internet-delivered CBT to be equivalent to those of face-to-face CBT for psychiatric and somatic disorders [25].

Over the past decade, a number of telemedicine interventions, smartphone apps, and websites have been created to help users develop good sleep habits and improve sleep quality through sleep monitoring, sleep hygiene, CBT-I, or mindfulness meditation [26-29]. Systematic reviews suggest that internet-based CBT-I has medium to large effects on sleep outcomes among youth and adults [30,31]. Meta-analyses indicate that digitally delivered CBT and telemedicine-based CBT are noninferior to face-to-face CBT [32,33]; however, only a small number of randomized controlled trials (RCTs) directly comparing 2 treatments were available to be included to pool the effects (n=4 and n=2). A recent systematic review of mobile phone sleep interventions demonstrated the effectiveness of mobile health technologies for improving sleep [34]. However, these reviews covered a single psychosocial intervention (eg, CBT-I) or particular eHealth modality (eg, mobile devices). We found no systematic review or meta-analysis summarizing and comparing the effects of multiple eHealth-based psychosocial interventions for insomnia. In addition, more novel trials with robust study designs and large sample sizes have been published in the past 5 years that were not included in the previous reviews [12,13,26,35-39]. Hence, an updated systematic review is warranted to examine the effectiveness of eHealth as a treatment for adults with insomnia.

### Objectives

The primary purpose of this systematic review was to summarize the evidence of the effectiveness of eHealth-based psychosocial interventions on insomnia symptoms, including insomnia severity and sleep quality. We investigated the effectiveness of eHealth-based psychosocial interventions compared with inactive controls and in-person comparators. Subgroup and metaregression analyses were performed to identify whether and to what extent population and intervention characteristics were related to treatment effectiveness on insomnia symptoms. As secondary outcomes, we evaluated the effects of eHealth-based psychosocial interventions on improving sleep diary parameters and mental health–related outcomes, for instance, sleep efficiency, maladaptive beliefs about sleep, and fatigue and depression symptoms.

## Methods

This systematic review and meta-analysis were conducted in accordance with the PRISMA (Preferred Reporting Items for Systematic Reviews and Meta-Analyses) reporting guidelines [40]. This study was registered in PROSPERO (CRD42021233241).

### Search Strategy

A systematic search was performed in the following databases—PubMed, Embase, Web of Science, PsycINFO, and the Cochrane Central Register of Controlled Trials—from inception to February 16, 2021, with PubMed searched up to December 6, 2021. Our search strategy combined index terms and text words associated with insomnia, eHealth, and intervention (the full list of search terms is provided in Multimedia Appendix 1). We also searched the gray literature, including dissertations, clinical trial registries, and conference proceedings, for unpublished studies. Furthermore, we manually scanned the references of relevant studies and reviews to identify any additional studies of relevance.

### Eligibility Criteria and Study Selection

The search strategy and selection criteria were developed using the Population, Intervention, Comparison, Outcomes, and Study Design framework [41]. Studies were eligible if they assessed eHealth-based psychosocial interventions with the primary aim of improving insomnia symptoms; detailed information on inclusion and exclusion criteria is provided in Textbox 1. After identifying and removing duplicates, 2 reviewers (WD and JW) performed the screening, with disagreements resolved through discussion with a third reviewer (RK).

**Inclusion and exclusion criteria for this study.**

- Inclusion criteria
  Participants: participants were adults with Diagnostic and Statistical Manual of Mental Disorders– or International Classification of Sleep Disorders–diagnosed insomnia or self-reported insomnia complaints.
  Intervention: “*eHealth-based psychosocial interventions*” included psychosocial treatments that were delivered via computers (email or websites), mobile phones (apps or SMS text messages and phone calls), telemedicine, digital games, and tablets or related technologies [1]. Blended interventions for insomnia, which combine little face-to-face care with the intervention primarily via eHealth channels [14], were also included if the eHealth component constituted ≥75% of the intervention sessions or the core of the intervention was eHealth-based [30].
  Comparison: we only included studies with “in-person” controls or “inactive” controls.
    “In-person” meant face-to-face psychotherapy, for instance, cognitive behavioral therapy.
Control groups classified as “inactive” were those in which participants were put on a waiting list or placed in a placebo group, for instance, usual care or sleep hygiene [4,5].
  Outcome measures: we included studies that had at least one insomnia severity– or sleep quality–related outcome measure.
  Study design: we included randomized controlled trials.
- Exclusion criteria
  We excluded studies that included participants with sleep disorders other than insomnia (eg, obstructive sleep apnea or narcolepsy), with specific medical conditions (eg, epilepsy, chronic pain, cancer, or recent surgery), and belonging to certain special populations (eg, overnight shift workers and pregnant or puerperium women).
  We further excluded trials using smart devices solely to record sleep data or physical therapy.
  Trials without a control group or noninferiority trials comparing 2 eHealth interventions were excluded.

### Data Analysis

Data were extracted in duplicate (WD and JW) using systematic extraction forms. We recorded the following information: study design, study sample (age, sex, sample size, diagnostic information, and relevant inclusion criteria), intervention (name, type, delivery mode, duration, functionality, and features of the eHealth-based psychosocial interventions), comparison conditions, and study outcomes. The delivery mode of the eHealth-based psychosocial interventions was classified as phone-delivered (eg, phone calls, apps, or SMS text messages on a smartphone or telephone), computer-assisted (eg, using a computer to log into a website, dashboard, or video chat room), or mixed mode (eg, an intervention delivered using both computer-assisted and phone-delivered components or an intervention that only stated the use of the internet or website). We also classified the functions of eHealth as informing, instructing, displaying, guiding, reminding, and communicating [42]. For studies with overlapping data sets, we used the most recent study with relevant outcome measure data. If the data were abstracted or unclear, we contacted the corresponding author by email for clarification. If the author did not respond after 2 contact attempts, we excluded the study. Disagreements between reviewers regarding data abstraction were resolved through discussion with a third reviewer. Interrater reliability between the 2 reviewers, assessed using the Cohen κ, indicated acceptable agreement (Cohen κ=0.86) [43].

A random-effects model was used in the meta-analysis, which allows for subtle differences across studies because of variability in sampling or treatment [44]. The Hedges *g* was used to estimate the effect size by pooling the mean difference of the continuous measures between the intervention and the control condition, which is the unbiased standard mean difference [45,46]. For multi-arm studies including 2 eHealth-based psychosocial interventions, we included each pairwise comparison separately by evenly dividing the shared control condition among the comparisons [47]. For studies comparing an eHealth-based psychosocial intervention with both in-person and inactive control conditions, corresponding comparisons were used in the meta-analyses for the different conditions. We presented pooled results using forest plots. Between-study heterogeneity was estimated using the Cochran *Q* and *I*^2^ statistics. Heterogeneity was interpreted according to the following thresholds: low (0%-40%), moderate (30%-60%), substantial (50%-90%), and considerable (75%-100%).

The primary outcomes were improvement in insomnia severity and sleep quality as frequently measured using the Insomnia Severity Index and the Pittsburgh Sleep Quality Index, respectively. Preplanned analyses of inactive and in-person comparison conditions on 2 primary outcomes were performed separately to examine whether eHealth-based psychosocial interventions were more effective than inactive controls and calculate the difference between eHealth-based psychosocial interventions and in-person controls. In addition, we conducted exploratory subgroup analyses and metaregression analyses on only those studies that compared eHealth-based psychosocial interventions with inactive controls. We did this to examine whether population or eHealth intervention aspects may affect the effectiveness of the interventions on insomnia symptom change and to exclude sources likely to be highly heterogeneous. The following study characteristics and intervention features were assessed: population (clinically diagnosed insomnia or subclinical insomnia), eHealth delivery mode (phone-delivered, computer-assisted, or mixed mode), therapeutic approach (eHealth-based CBT [eCBT] or non-CBT), guidance modality (guided by a trained therapist, guided by an animated therapist, or no guidance), tailored feedback provided (yes or no), reminder or encouragement provided (yes or no), baseline insomnia severity, sleep medication, intervention duration (in weeks and sessions), number of intervention components, and number of eHealth functions.

The secondary outcomes of interest were changes in sleep parameters derived from sleep diaries and improvements in mental health–related outcomes, including sleep onset latency (SOL), total sleep time (TST), wake after sleep onset (WASO), number of nocturnal awakenings (NWAK), sleep efficiency, maladaptive beliefs about sleep, fatigue, depression symptoms, anxiety symptoms, and quality of life. Outcomes reported in ≥2 studies were pooled separately.

We conducted influence analysis on all studies via the “*leave-one-study-out meta-analysis*” method recalculating the pooled effect sizes after removing each study to detect potential outliers [48]. Publication bias was examined graphically using funnel plots. The degree of asymmetry was tested using the Egger regression test of the intercept with a 1-tailed significance level of α=.05 applied to the primary and secondary outcome analyses [49]. Furthermore, the trim-and-fill analysis by Duval and Tweedie [50] was applied to adjust the effect size for missing studies. All statistical analyses were conducted using Stata (version 14.1; StataCorp) [51].

### Quality Assessment

The Cochrane risk-of-bias tool for randomized trials was used to assess the methodological quality of the RCTs [52]. This tool examines 5 domains of trial design: the risk of bias in *the randomization process*, *deviations from the intended interventions* *(effect of assignment to intervention)*, *missing outcome data*, *measurement of the outcome*, and *selection of the reported result*, ranking each domain as high, low, or with some concerns for risk of bias. Studies with a high risk of bias in at least one domain were rated as having a high overall risk of bias. In total, 2 reviewers (WD and JW) evaluated the risk of bias for each domain independently, with good interrater agreement (κ=0.70). Disagreements between reviewers were resolved through discussion with a third reviewer (RK).

## Results

### Study Selection

**Figure 1:**
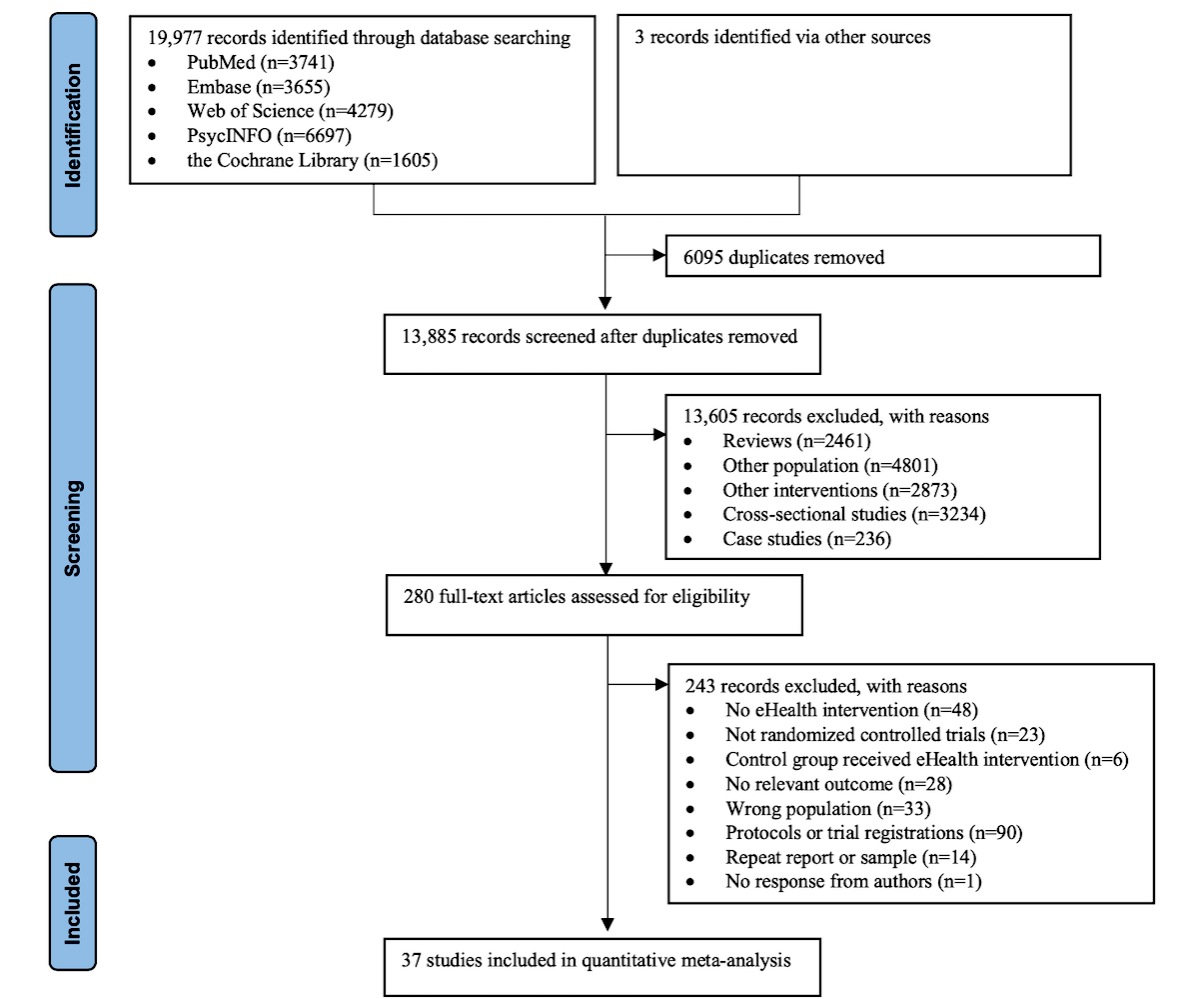
PRISMA (Preferred Reporting Items for Systematic Reviews and Meta-Analyses) flowchart of study selection.

The systematic literature search identified 19,980 records (including additional searching), of which 37 studies were deemed eligible and included in the quantitative meta-analysis (for the study selection process; Figure 1) [12,13,26,35-39,53-81]. Of the included studies, most RCTs (28/37, 76%) were 2-arm trials, 22% (8/37) were 3-arm trials (4/8, 50% of the studies included 2 eHealth-based psychosocial intervention groups and 1 control group [35,36,63,65], and 4/8, 50% of the studies included 1 eHealth-based psychosocial intervention group and 2 control groups [59,66,67,78]), and 3% (1/37) were 4-arm trials including 2 eHealth-based psychosocial interventions and 2 control groups [13]. Altogether, these trials described 42 eHealth-based psychosocial interventions that were combined into 46 unique pairwise comparisons on which our statistical analyses were based (for study characteristics, see Table 1).

**Table 1:** Study characteristics (N=37).

Study, year, and country^a^	Sample type	Severity (baseline)	Sleep medications	Age (years), mean (SD)	Sample size; female participants (%)^b^	Intervention	Control condition
Arnedt et al [26], 2020, United States	ICSD-3^c^ chronic insomnia	ISI^d^ >13.8	26 (78.8%); 25 (78.1%)	47.2 (16.3)	65 (IG^e^: 33; CG^f^: 32); 46 (71)	SleepTM (eCBT-I^g^)	Face-to-face CBT^h^
Bedford et al [53], 2018, United States	Self-reported insomnia and mild depression	ISI of approximately 14	NS^i^	32.7 (7.5)	24 (IG: 12; CG: 12); 5 (21)	ePST^j^	Minimal-contact control
Bernstein et al [54], 2017, United States	Self-reported insomnia	ISI >15	NS	54.3 (12.6)	88 (IG: 43; CG: 45); 74 (84.1)	Go! to Sleep (eCBT-I and general stress management techniques)	Waitlist
Blom et al [55], 2015, Sweden	Self-reported insomnia	ISI >10	14 (58%); 16 (67%)	56.1 (10.2); 52.6 (16.6)	48 (IG: 24; CG: 24); 23 (48)	eCBT-I	Group CBT
Chao et al [39], 2021, United States	DSM-5^k^ criteria for insomnia	NS	4 (10%); 9 (20%)	54 (6)	85 (IG: 39; CG: 46); 21 (24.7)	eCBT-I	Waitlist
Cheng et al [56], 2019, United States	DSM-5 criteria for insomnia	ISI >13	NS	44.5 (15.8); 45.7 (15.1)	658 (IG: 358; CG: 300); 518 (79)	Sleepio program (eCBT-I)	Sleep education
Christensen et al [57], 2016, Australia	Morin modified diagnostic insomnia	NS	NS	42.5 (12.2)	1149 (IG: 574; CG: 575); 845 (74)	SHUTi^l^ (eCBT-I)	Sleep education
Ebert et al [58], 2015, United States	Self-reported insomnia	ISI ≥15	NS	48.5 (9.9)	128 (IG: 64; CG: 64); 95 (74.2)	GET.ON Recovery (eCBT^m^)	Waitlist
Espie et al [59], 2012, United Kingdom	DSM-5 criteria for insomnia	Sleep efficiency ≤79%	10 (18.2%); 15 (27.8%); 8 (14.5%)	49 (13.5)	164 (IG: 55; CG 1: 54; CG 2: 55); 120 (73)	Sleepio program (eCBT-I)	CG 1: TAU^n^; CG 2: imagery relief therapy plus TAU
Espie et al [12], 2019, United Kingdom	Self-reported insomnia	8-item SCI^o^ ≤16	NS	48.4 (13.9); 47.7 (13.6)	1711 (IG: 853; CG: 858); 1329 (78)	Sleepio program (eCBT-I)	Sleep hygiene education plus TAU
Freeman et al [60], 2017, United Kingdom	Self-reported insomnia	8-item SCI ≤16	55 (3%); 51 (3%)	24.8 (7.7); 24.6 (7.6)	3755 (IG: 1891; CG: 1864); 2676 (71)	Sleepio program (eCBT-I)	TAU
Glozier et al [61], 2019, Australia	Diagnostic criteria for insomnia and depression	QIDS-SR^p^ ≥8 and ISI ≥8	NS	58.6 (6.3); 58.1 (6.1)	87 (IG: 45; CG: 42); 0 (0)	SHUTi (eCBT-I and adjunctive in-person treatment for depression)	Sleep education
Hagatun et al [62], 2018, Norway	Diagnostic criteria for insomnia	NS	NS	44.9 (13)	181 (IG: 95; CG: 86); 121 (67)	SHUTi (eCBT-I)	Sleep education
Ho et al [63], 2014, China	Self-reported insomnia	ISI ≥10	24 (23.3%); 28 (26.9%); 27 (25.7%)	38.5 (12.5)	312 (IG 1: 103; IG 2: 104; CG: 105); 222 (71)	IG 1: eCBT-I with telephone support; IG 2: eCBT-I without support	Waitlist
Horsch et al [64], 2017, the Netherlands	Self-reported insomnia	ISI ≥7	8 (11%); 3 (4%)	39 (13); 41 (13.9)	151 (IG: 74; CG: 77); 94 (49.3)	Sleepcare (eCBT-I)	Waitlist
Krieger et al [65], 2019, Switzerland	ICSD-3 criteria for insomnia	ISI of approximately 17	8 (19%); 4 (9.8%); 2 (9.5%);	42.2 (12.4)	104 (IG 1: 42; IG 2: 41; CG: 21); 71 (68.3)	IG 1: multicomponent internet-based guided treatment; IG 2: sleep restriction	TAU
Lancee et al [66], 2012, the Netherlands	DSM-4^q^ criteria for insomnia	SLEEP-50≥19 with sleep efficiency <85%	20 (9.3%); 17 (8.4%); 11 (5.5%)	51.8 (12.1)	623 (IG: 216; CG 1: 202; CG 2: 205); 520 (67.7)	eCBT-I	CG 1: waitlist; CG 2: paper-and-pencil self-help CBT via mail
Lancee et al [67], 2016, the Netherlands	DSM-5 criteria for insomnia	ISI ≥10	11 (36.7%); 8 (26.7%); 9 (30%)	41.2 (14.1); 38.5 (13.1); 45.1 (13.7)	90 (IG: 30; CG 1: 30; CG 2: 30); 73 (81)	eCBT-I	CG 1: face-to-face CBT; CG 2: waitlist
Lorenz et al [68], 2019, Switzerland	Self-reported insomnia	ISI ≥8	9 (31%); 11 (41%)	41.7 (17.3); 44 (20.1)	56 (IG: 29; CG: 27); 39 (70)	Mementor somnium (eCBT-I)	Waitlist
McCurry et al [69], 2016, United States	Self-reported insomnia	ISI ≥12	NS	55.0 (3.5); 54.7 (4.7)	106 (IG: 53; CG: 53); 106 (100)	Telephone-based CBT-I^r^ (first session inviting in person at the office)	Menopause education
McGrath et al [70], 2017, Ireland	Self-reported insomnia	PSQI^s^ ≥6	NS	59.7 (9.9); 58.3 (11.9)	134 (IG: 67; CG: 67); 82 (61.2)	Sleepio program (eCBT-I) and group education	Cardiovascular risk factor education
Okajima et al [13], 2020, Japan	Self-reported insomnia	ISI ≥8	NS	42.7 (11.5)	92 (IG 1: 24; IG 2: 23; CG 1: 23; CG 2: 22); 32 (35)	IG 1: tailored brief behavioral therapy for insomnia; IG 2: standard brief behavioral therapy	CG 1: self-monitoring; CG 2: waitlist
Paivi et al [71], 2019, Finland	Self-reported insomnia	ISI ≥8	21 (48.8%); 16 (40%)	53.5 (13.4)	83 (IG: 43; CG: 40); 53 (63.9)	Internet-delivered acceptance and commitment therapy	Waitlist
Pillai et al [72], 2015, United States	DSM-5–diagnosed insomnia	NS	NS	49.8 (13.5)	22 (IG: 13; CG: 9); 14 (63)	Sleepio (eCBT-I)	Sleep education
Rayward et al [35], 2020, Australia	Self-reported insomnia	PSQI ≥5	NS	52 (6.9)	275 (IG 1: 110; IG 2: 110; CG: 55); 228 (83)	IG 1: mHealth^t^ physical activity and sleep health (the Balanced app); IG 2: sleep health only (the Balanced app)	Waitlist
Ritterband et al [73], 2009, United States	DSM-4 criteria for insomnia	ISI ≥14	6 (27.3%); 9 (40.9%)	44.9 (11)	44 (IG: 22; CG: 22); 34 (77)	SHUTi (eCBT-I)	Waitlist
Ritterband et al [74], 2017, United States	DSM-4 criteria for insomnia	ISI ≥13	NS	43.3 (11.6)	303 (IG: 151; CG: 152); 218 (71.9)	SHUTi (eCBT-I)	Sleep education
Sato et al [75], 2019, Japan	DSM-5 criteria for insomnia	PSQI >5.5	NS	49.4 (13.8); 50.5 (8.8)	23 (IG: 11; CG: 12); 18 (78)	eCBT-I	TAU
Ström et al [76], 2004, Sweden	DSM-4 criteria for insomnia	Experienced sleep problems for a mean duration of 10.6 years	47 (43.2%)	44.1 (12)	109 (IG: 54; CG: 55); 71 (65)	eCBT-I	Waitlist
Sunnhed et al [36], 2020, Sweden	DSM-5 criteria for insomnia	ISI ≥11	29 (40.3%); 34 (46.6%); 30 (40.5%)	51.5 (12.5); 51.8 (14.5); 54.2 (14.6)	219 (IG 1: 72; IG 2: 73; CG: 74); 160 (73)	IG 1: cognitive therapy; IG 2: behavioral therapy	Waitlist
Suzuki et al [77], 2008, Japan	Self-reported insomnia	PSQI >4	1 (5%); 1 (4.8%)	39.6 (8.2)	43 (IG: 21; CG: 22); 16 (39)	eCBT-I	Waitlist
Taylor et al [78], 2017, United States	Diagnosis of chronic insomnia	NS	10 (29%); 2 (6%); 5 (15%)	32.7 (7.7)	100 (IG: 34; CG 1: 33; CG 2: 33); 17 (17)	eCBT-I	CG 1: in-person CBT-I; CG 2: minimal-contact control
Van der Zweerde et al [79], 2019, the Netherlands	DSM-5 criteria for insomnia and with subclinical depression	ISI >10; PHQ-9^u^ >4	NS	44.6 (13.1); 46.3 (15.1)	104 (IG: 52; CG: 52); 85 (82)	i-Sleep (eCBT-I)	Sleep diary monitoring only
Van der Zweerde et al [37], 2020, the Netherlands	DSM-5 criteria for insomnia	ISI >15	23 (33%); 25 (39%)	51.7 (15.8); 49.4 (16.01)	134 (IG: 69; CG: 65); 87 (65)	i-Sleep (eCBT-I)	TAU
Van Straten et al [80], 2014, the Netherlands	DSM-5 criteria for insomnia	PSQI of approximately 12	36 (30.5%)	49.4 (12.9)	118 (IG: 59; CG: 59); 83 (70.3)	eCBT-I	Waitlist
Vedaa et al [38], 2020, Norway	Self-report of insomnia	ISI ≥12	480 (55.3%); 514 (60.3%)	45 (14)	1721 (IG: 868; CG: 853); 1167 (68)	SHUTi (eCBT-I)	Sleep education
Vincent and Lewycky [81], 2009, Canada	DSM-5 criteria for insomnia	ISI of approximately 18	NS	NS	118 (IG: 59; CG: 59); 79 (66.9)	eCBT-I	Waitlist

^a^The studies by Espie et al [59], Lancee et al [66,67], Ho et al [63], Krieger et al [65], Okajima et al [13], Rayward et al [35], and Sunnhed et al [36] used a multi-arm study design.

^b^Sample size at baseline reported separately for the intervention and control groups.

^c^ICSD-3: International Classification of Sleep Disorders–Third Edition.

^d^ISI: Insomnia Severity Index.

^e^IG: intervention group.

^f^CG: control group.

^g^eCBT-I: eHealth-based cognitive behavioral therapy for insomnia.

^h^CBT: cognitive behavioral therapy.

^i^NS: not specified in the study.

^j^ePST: eHealth-based problem-solving treatment.

^k^DSM-5: Diagnostic and Statistical Manual of Mental Disorders, Fifth Edition.

^l^SHUTi: Sleep Healthy Using the Internet.

^m^eCBT: eHealth-based CBT.

^n^TAU: treatment as usual.

^o^SCI: Sleep Condition Indicator.

^p^QIDS-SR: Quick Inventory of Depressive Symptomatology.

^q^DSM-4: Diagnostic and Statistical Manual of Mental Disorders, Fourth Edition.

^r^CBT-I: CBT for insomnia.

^s^PSQI: Pittsburgh Sleep Quality Index.

^t^mHealth: mobile health.

^u^PHQ-9 Patient Health Questionnaire-9.

### Characteristics of the Included Studies

The included studies were published between 2004 and 2021 and were conducted in the United States (11/37, 30%) [26,39,53,54,56,58,69,72,73,78], the Netherlands (6/37, 16%) [37,64,66,67,79,80], Australia (3/37, 8%) [35,57,61], Japan (3/37, 8%) [13,75,77], Sweden (3/37, 8%) [36,55,76], the United Kingdom (3/37, 8%) [12,59,60], Norway (2/37, 5%) [38,62], Switzerland (2/37, 5%) [65,68], Canada (1/37, 3%) [81], China (1/37, 3%) [63], Ireland (1/37, 3%) [70], and Finland (1/37, 3%) [72]. The sample sizes ranged from 22 to 3755 (total sample size=13,227). The total sex distribution was skewed toward female participants (9442/13,227, 71%) and male participants (3785/13,227, 29%). The average age of the participants ranged from 24.6 to 58.6 years, with a mean of 46.3 (SD 5.6) years. Approximately half (20/37, 54%) of the studies included participants with a clinical diagnosis of insomnia, with the remainder (17/37, 46%) including people with self-reported insomnia symptoms. A total of 3% (1/37) of the studies only included female participants as they examined peri- or postmenopausal insomnia [69], with another study including only male participants with insomnia and depression symptoms [61]. Of the included studies, 86% (32/37) provided eHealth-based CBT-I as the intervention arm. Other studies (5/37, 14%) used eHealth-based problem-solving treatment [53], behavioral therapy [13,36,65], cognitive therapy [36], key behavior change techniques [35], and self-help acceptance and commitment therapy [71]. In total, 3 studies focused on blended interventions, 1 combining adjunctive in-person treatment for depression [61] and the other 2 conducting the first session at the office [69,70]. In total, 11% (4/37) of the studies compared eCBT with face-to-face CBT [26,55,67,70], with the rest reporting comparisons with inactive controls.

The delivery mode of the eHealth interventions ranged from mixed-mode interventions (20/37, 54%) to computer-assisted interventions (13/37, 35%) and phone-delivered interventions (5/37, 14%). Sleep Healthy Using the Internet [82] and Sleepio ([83]; Big Health Ltd) were frequently used eHealth programs, each appearing in 16% (6/37) of the studies. i-Sleep [84] was also used quite frequently. Regarding the guidance modality, 46% (17/37) of the included studies reported that the eHealth intervention was instructed by a trained human therapist, for example, under the guidance of an expert clinician or trained coach in vivo. A total of 19% (7/37) of the studies reported that the participants were guided by an animated therapist, with the remainder (15/37, 41%) reporting no guidance. Regarding feedback, the eHealth interventions in 86% (32/37) of the studies provided tailored feedback, including feedback on the web using real-time user data such as personal summary statistics, progress scores, or automated individual advice. In contrast, a small proportion of the studies (7/37, 19%) did not provide tailored feedback. Notably, there were 5% (2/37) of the studies comprising 2 separate eHealth intervention arms, one with tailored feedback and the other without tailored feedback [13,59]; both concluded that tailored feedback could enhance the efficacy. In addition, most of the eHealth interventions in the included studies (34/37, 92%) reminded or encouraged participants to stay involved via email, SMS text message, or phone call, whereas only a minority (5/37, 14%) did not mention the use of any reminder or encouragement; 5% (2/37) of the studies included 2 eHealth arms, where only 1 arm set reminders [13,59] (see Table 2 for the intervention characteristics). The duration of the interventions ranged from 2 to 12 weeks, with a mean of 7.05 (SD 2.24) weeks, and the average number of treatment sessions was 6.2 (SD 1.0) ranging from 2 to 8 sessions, with durations of 20 to 60 minutes per session. Questionnaires and sleep diaries were used to evaluate the effectiveness of the eHealth interventions in improving insomnia symptoms, sleep status, or mental health. Detailed information on the outcome indicators and assessments can be found in Table 3. On average, the percentage of participants in the eHealth intervention groups that completed the postintervention assessment was 74.6% (SD 18.8%). The follow-up duration ranged from 3 weeks to 12 months, with a mean of 5.6 (SD 3.1) months. Multimedia Appendix 2 [12,13,26,35-39,53-81] includes a summary of the full details of the included studies and information on process outcomes.

**Table 2:** eHealth-based psychosocial intervention characteristics (N=37).

Study, year	Delivery mode	Intervention details	Intervention components	Functionality of eHealth	Sessions, n	Weeks, n
Arnedt et al [26], 2020	Computer-assisted	An expert clinician delivered the intervention according to clinical practice guidelines for CBT^a^ for insomnia.	SHE^b^, SCT^c^, SRT^d^, CT^e^, RS^f^, CW^g^, and RP^h^	Inform, instruct, record, display, guide, remind, and communicate	6	6
Bedford et al [53], 2018	Computer-assisted	Tailored intervention; guided by a supportive animated therapist and provided tailored feedback; with “homework” activities and session summary	(1) Defining the problem, (2) goal setting, (3) brainstorming solutions, (4) selecting solutions, (5) action planning, and (6) evaluating the success and troubleshooting	Inform, instruct, record, display, guide, remind, and communicate	6	8
Bernstein et al [54], 2017	Mixed methods	Interactive web-based lessons with daily email reminders	SHE, SCT, SRT, CT, RS, SM^i^, and MT^j^	Inform, instruct, record, display, remind, and communicate	6	6
Blom et al [55], 2015	Mixed methods	Mobile phone SMS text message or phone call to remind and encourage by experienced therapist; intervention consisted of text to read, questions to answer on theory, behavioral assignments, worksheets, and a sleep diary	SHE, SCT, SRT, MT, CR^k^, SM, and RP	Inform, instruct, record, display, guide, remind, and communicate	8	8
Chao et al [39], 2021	Phone-delivered	CBT-I^l^ consists of several sessions of sleep restriction, stimulus control, and cognitive restructuring related to sleep concerns and administered via telephone by trained therapists.	SRT, SCT, and CR	Inform, instruct, record, display, guide, and communicate	8	8
Cheng et al [56], 2019	Mixed methods	Animated therapist guide; support and prompts and reminders by email and SMS text message; tailored feedback from individual diary data; access to a library with background information, a forum with other users of the program, a user case file, and weekly live expert sessions	SHE, SRT, SCT, CR, PI^m^, and RS	Inform, instruct, record, display, guide, remind, and communicate	6	12
Christensen et al [57], 2016	Mixed methods	Unguided and self-help intervention with automated reminders; interactivity and personalized feedback	SHE, SRT, SCT, CR, and RP	Inform, instruct, record, display, and remind	6	6
Ebert et al [58], 2015	Mixed methods	Supported by trained coaches according to a manual; reminders; feedback on homework assignments via a messaging system	SHE, SCT, SRT, BT^n^, GJ^o^, MCT^p^, and PL^q^	Inform, instruct, record, display, guide, remind, and communicate	6	8
Espie et al [59], 2012	Mixed methods	Animated therapist guide; support, prompts, or reminders by email; tailored feedback from individual diary data	SHE, SRT, SCT, CR, PI, RS, MT, and IM^r^	Inform, instruct, record, display, guide, remind, and communicate	6	6
Espie et al [12], 2019	Mixed methods	Animated therapist guide; support, prompts, or reminders by email and SMS text message; tailored feedback from individual diary data; access to a library with background information, a forum with other users of the program, and a user case file	SHE, SRT, SCT, CR, PI, RS, MT, and IM	Inform, instruct, record, display, guide, remind, and communicate	6	12
Freeman et al [60], 2017	Mixed methods	Animated therapist guide; support, prompts, or reminders by email and SMS text message; tailored feedback from individual diary data; access to a library with background information, a forum with other users of the program, and a user case file	SHE, SRT, SCT, CR, PI, RS, MT, and IM	Inform, instruct, record, display, guide, remind, and communicate	6	10
Glozier et al [61], 2019	Mixed methods	Unguided; automated midweek reminders to enter sleep diaries, implement strategies, and commence the next module were also sent; tracking of individual completion of the programs	SHE, SRT, SCT, CR, and RP	Inform, instruct, record, display, and remind	6	12
Hagatun et al [62], 2018	Mixed methods	Unguided and self-help intervention with automated reminders; interactivity and personalized feedback	SHE, SRT, SCT, CR, and RP	Inform, instruct, record, display, and remind	6	9
Ho et al [63], 2014	IG^s^ 1: mixed methods; IG 2: computer-assisted	Provided text to read weekly together with some diagrams and a 15-minute audio clip on relaxation training; participants in IG 1 received weekly telephone support from therapist; participants in IG 2 did not receive support	IG 1/2: SHE, SRT, SCT, CR, RS, and RP	IG 1: inform, instruct, record, display, guide, remind, and communicate; IG 2: inform, instruct, record, and display	NS^t^	7
Horsch et al [64], 2017	Phone-delivered	Fully automated and without any input from therapists; automatic warnings were built in when participants slept for <5 hours on average. The app interacted with the participants via dialogs on the conversation screen.	SHE, RS, SRT, and PS^u^	Inform, instruct, record, display, remind, and communicate	NS	6-7
Krieger et al [65], 2019	IG 1/2: mixed methods	IG 1: guided by trained therapist and provided feedback and reminders; IG 2: mainly consisted of sleep restriction instructions that were embedded in an introductory and psychoeducational module, same guidance as IG 1	IG 1: SHE, SRT, SCT, CR, RP, RS, and SM; IG 2: SRT	IG 1/2: inform, instruct, record, display, guide, remind, and communicate	8	8
Lancee et al [66], 2012	Computer-assisted	A simple website that did not include interaction or individual tailoring; no email support from a therapist; provided email reminders for assessment	SHE, SRT, SCT, CR, and PI	Inform, instruct, record, display, and remind	6	6
Lancee et al [67], 2016	Computer-assisted	Guided by trained coach, provided reminders and personal feedback by Master’s-level students of psychology	SHE, SRT, SCT, CR, and PI	Inform, instruct, record, display, guide, remind, and communicate	6	8
Lorenz et al [68], 2019	Computer-assisted	Guided by an animated sleep coach; feedback based on the sleep diary data	SHE, SRT, SCT, CR, RP, and RS	Inform, instruct, record, display, and communicate	6	6
McCurry et al [69], 2016	Phone-delivered	Guided by trained coaches; provided information about age-related sleep changes, sleep hygiene, sleep restriction, and stimulus control procedure	SHE, SRT, PL, CR, CW, and RP	Inform, instruct, record, display, guide, and communicate	6	8
McGrath et al [70], 2017	Mixed methods	Animated therapist guide; support, prompts, or reminders by email and SMS text message; tailored feedback from individual diary data; access to a library with background information, a forum with other users of the program, and a user case file	SHE, SRT, SCT, CR, and RS	Inform, instruct, record, display, guide, remind, and communicate	6	6-8
Okajima et al [13], 2020	IG 1/2: phone-delivered	IG 1: guided by an expert in sleep science, provided reminders and additional suggestions, and included individually tailored challenge tasks and sleep-related articles; IG 2: unguided, no tailored feedback	IG 1/2: SHE, SCT, SRT, and RS	IG 1: inform, instruct, record, display, guide, remind, and communicate; IG 2: inform, instruct, record, and display	NS	2
Paivi et al [71], 2019	Computer-assisted	Self-help intervention with weekly email-based automated reminders	VA^v^, MT, CT, OA^w^, and PL	Inform, instruct, record, and remind	6	6
Pillai et al [72], 2015	Mixed methods	Animated therapist guide; support, prompts, or reminders by email; tailored feedback from individual diary data	SHE, SRT, SCT, CR, PI, RS, MT, and IM	Inform, instruct, record, display, guide, remind, and communicate	6	6
Rayward et al [35], 2020	IG 1/2: phone-delivered	IG 1/2: sleep intervention based on Social Cognitive Theory and operationalized BCTs^x^. The intervention implemented key BCTs, including self-monitoring, goal setting, and personalized feedback (including weekly reports, tool sheets, and prompts).	IG 1: SHE, BCT, and PA^y^; IG 2: SHE and BCT	IG 1/2: inform, instruct, record, display guide, remind, and communicate	NS	12
Ritterband et al [73], 2009	Mixed methods	Unguided; automated reminders to complete diary entries were sent daily via email; interactivity and personalized feedback	SHE, SRT, SCT, CT, and RP	Inform, instruct, record, display, and remind	6	9
Ritterband et al [74], 2017	Mixed methods	Unguided; automated reminders to complete diary entries were sent daily via email; interactivity and personalized feedback	SHE, SRT, SCT, CT, and RP	Inform, instruct, record, display, and remind	6	9
Sato et al [75], 2019	Computer-assisted	Guided by a cognitive behavioral therapist; weekly emails to ask participants about their homework and progress	SHE, SRT, SCT, CT, and RP	Inform, instruct, record, display, guide, remind, and communicate	5	6
Ström et al [76], 2004	Computer-assisted	Guided by trained therapists, provided email reminders and feedback for monitoring of homework assignments	SHE, SRT, SCT, CT, and RS	Inform, instruct, record, guide, remind, and communicate	NS	5
Sunnhed et al [36], 2020	IG 1/2: mixed methods	Both interventions based on the web-based platform; guided by trained therapists; offered 15-minute weekly telephone support consisting of feedback on registered homework	IG 1: (1) sleep-interfering or sleep-related worry, (2) unhelpful beliefs about sleep, (3) attentional bias and monitoring for sleep-related threat, (4) misperception of sleep, and (5) safety behaviors; IG 2: SHE, SRT, and SCT	IG 1/2: inform, instruct, record, display, and communicate	10	10
Suzuki et al [77], 2008	Mixed methods	Unguided, the website provided automatically positive encouragement, weekly summary, and advice	SHE, RS, CT, SRT, and RW^z^	Inform, instruct, record, display, remind, and communicate	NS	2
Taylor et al [78], 2017	Computer-assisted	Unguided, provided reminders for sleep diary and tailored recommendations for the sleep restriction; interactive components were included, such as games, quizzes, and prompts	SHE, SRT, SCT, CT, and RS	Inform, instruct, record, remind, and communicate	NS	6
Van der Zweerde et al [79], 2019	Computer-assisted	Guided by trained clinical psychology graduate students, provided feedback on exercise sleep data based on the diary and motivated participants to persevere in the treatment	SHE, SRT, SCT, CR, RS, and RP	Inform, instruct, record, remind, display, guide, and communicate	5	8
Van der Zweerde et al [37], 2020	Computer-assisted	Guided by trained nurses, provided tailored feedback and encouragement	SHE, SRT, SCT, CR, RS, and RP	Inform, instruct, record, display, guide, remind, and communicate	5	8
Van Straten et al [80], 2014	Computer-assisted	Guided by trained coach, provided tailored feedback and encouragement	SHE, SRT, SCT, CR, RS, and RP	Inform, instruct, record, display, guide, remind, and communicate	6	6
Vedaa et al [38], 2020	Mixed methods	Unguided; automated reminders to complete diary entries were sent daily via email; feedback tailored to the user	SHE, SRT, SCT, CR, and RP	Inform, instruct, record, display, and remind	6	9
Vincent and Lewycky [81], 2009	Computer-assisted	Guided by a trained therapist, the main teaching component was present in an audiovisual mode with occasional text material appearing in the background to highlight particular points.	SHE, SRT, SCT, CR, and RS	Inform, instruct, record, display, guide, and communicate	5	5

^a^CBT: cognitive behavioral therapy.

^b^SHE: sleep hygiene education.

^c^SCT: stimulus control therapy.

^d^SRT: sleep restriction therapy.

^e^CT: cognitive therapy.

^f^RS: relaxation strategies.

^g^CW: constructive worry.

^h^RP: relapse prevention.

^i^SM: stress management.

^j^MT: mindfulness or meditation training.

^k^CR: cognitive reappraisal or reconstructuring.

^l^CBT-I: cognitive behavioral therapy for insomnia.

^m^PI: paradoxical intention.

^n^BT: boundary tactics.

^o^GJ: gratitude journal.

^p^MCT: metacognition techniques.

^q^PL: plan.

^r^IM: imagery.

^s^IG: intervention group.

^t^NS: not specified in the study.

^u^PS: persuasive strategies.

^v^VA: identify values and value-based actions.

^w^OA: observe and accept.

^x^BCT: behavior change technique.

^y^PA: physical activity.

^z^RW: reward.

**Table 3:** Summary of outcome indicators and assessment.

Study, year	Primary outcomes	Secondary outcomes	Proportion completing postintervention assessment, n (%)	Follow-up (month)
Arnedt et al [26], 2020	ISI^a^	Sleep diary, DBAS-16^b^, MFI-20^c^, PHQ-9^d^, GAD-7^e^, and SF-12^f^	IG^g^: 31 (94); CG^h^: 31 (97)	3
Bedford et al [53], 2018	ISI	Sleep diary, PHQ-9, and PCL-5^i^	IG: 7 (58); CG: 12 (100)	3
Bernstein et al [54], 2017	ISI	NS^j^	IG: 25 (58); CG: 35 (78)	2
Blom et al [55], 2015	ISI and SQ^k^	Sleep diary and MADRS-S^l^	IG: 17 (71); CG: 18 (75)	6
Chao et al [39], 2021	ISI and PSQI^m^	Sleep diary, FSS^n^, and HADS-D^o^	IG: 32 (82); CG: 39 (85)	6
Cheng et al [56], 2019	ISI	Sleep diary and QIDS^p^	NS	NS
Christensen et al [57], 2016	ISI	PHQ-9 and GAD-7	IG: 248 (43); CG: 333 (58)	6
Ebert et al [58], 2015	ISI and PSQI	Sleep diary and CES-D^q^	IG: 31 (48); CG: 51 (80)	6
Espie et al [59], 2012	SCI^r^	Sleep diary	IG: 43 (78); CG 1: 41 (75); CG 2: 47 (87)	2
Espie et al [12], 2019	SCI	Sleep diary, FSS, PHQ-9, and GAD-7	IG: 468 (55); CG: 517 (60)	4
Freeman et al [60], 2017	ISI	Sleep diary, PHQ-9, and GAD-7	IG: 733 (39); CG: 1142 (61)	4
Glozier et al [61], 2019	ISI	Sleep diary and CES-D	IG: 40 (89); CG: 35 (83)	6
Hagatun et al [62], 2018	ISI	Sleep diary	IG: 77 (81); CG: 65 (76)	6
Ho et al [63], 2014	ISI and PSQI	Sleep diary, DBAS^s^, MFI^t^, HADS-D, HADS-A^u^, and SF-36^v^	IG: 58 (56); CG 1: 61 (59); CG 2: 71 (68)	1; 3
Horsch et al [64], 2017	ISI and PSQI	Sleep diary, DBAS, CES-D, and HADS^w^	IG: 45 (61); CG: 62 (81)	3
Krieger et al [65], 2019	PSQI	DBAS, ADS-K^x^, and QoL^y^	IG 1: 37 (88); IG 2: 34 (83); CG: 20 (95)	6
Lancee et al [66], 2012	ISI	Sleep diary, CES-D, and HADS-A	IG: 168 (78); CG 1: 179 (87); CG 2: 184 (91)	4; 11
Lancee et al [67], 2016	SLEEP-50 and SQ	Sleep diary, CES-D, and HADS-A	IG: 15 (50); CG 1: 21 (70); CG 2: 23 (77)	3; 6
Lorenz et al [68], 2019	ISI	BDI^z^ and BSI-Anxiety^aa^	IG: 25 (93); CG: 27 (100)	12
McCurry et al [69], 2016	ISI	Sleep diary	IG: 47 (89); CG: 37 (70)	4
McGrath et al [70], 2017	ISI and SQ	Sleep diary, BDI, and BAI^ab^	IG: 34 (50); CG: 67 (100)	NS
Okajima et al [13], 2020	ISI	NS	IG 1: 20 (80); IG 2: 16 (70); CG 1: 22 (96); CG 2: 19 (86)	1; 3
Paivi et al [71], 2019	BNSQ^ac^	DBAS and BDI	IG: 41 (95); CG: 36 (90)	6
Pillai et al [72], 2015	ISI	Sleep diary and BAI	NS	NS
Rayward et al [35], 2020	PSQI	NS	IG 1: 102 (92.7); IG 2: 88 (80); CG: 50 (90.9)	3
Ritterband et al [73], 2009	ISI	Sleep diary	IG: 21 (96); CG: 22 (100)	6
Ritterband et al [74], 2017	ISI and SQ	Sleep diary	IG: 133 (88); CG: 142 (93)	6; 12
Sato et al [75], 2019	SQ	Sleep diary	IG: 11 (100); CG: 11 (92)	12
Ström et al [76], 2004	PSQI	Sleep diary and DBAS	IG: 28 (52); CG: 51 (93)	NS
Sunnhed et al [36], 2020	ISI	Sleep diary, HADS-D, HADS-A, and BBQ^ad^	IG: 68 (94); CG 1: 66 (90); CG 2: 74 (100)	6
Suzuki et al [77], 2008	PSQI	Sleep diary and K6^ae^	IG: 12 (57); CG: 18 (82)	0.75
Taylor et al [78], 2017	ISI and SQ	Sleep diary and DBAS	IG: 27 (79); CG 1: 30 (91); CG 2: 29 (88)	6
Van der Zweerde et al [79], 2019	ISI	Sleep diary, FSS, PHQ-9, and HADS-A	IG: 45 (87); CG: 47 (90)	3; 6
Van der Zweerde et al [37], 2020	ISI and SQ	Sleep diary, FSS, HADS-D, and HADS-A	IG: 43 (62); CG: 41 (63)	6; 12
Van Straten et al [80], 2014	PSQI	Sleep diary, CES-D, and QoL-VAS^af^	IG: 37 (63); CG: 45 (76)	2
Vedaa et al [38], 2020	ISI	Sleep diary, CFQ^ag^, and SF-12	IG: 584 (67); CG: 534 (63)	NS
Vincent and Lewycky [81], 2009	ISI and SQ	Sleep diary, DBAS, and MFI	IG: 40 (68); CG: 39 (66)	1

^a^ISI: Insomnia Severity Index.

^b^DBAS-16: Dysfunctional Beliefs and Attitudes about Sleep–16.

^c^MFI-20: Multidimensional Fatigue Inventory–20.

^d^PHQ-9: Patient Health Questionnaire–9.

^e^GAD-7: General Anxiety Disorder–7.

^f^SF-12: 12-item Short Form Survey.

^g^IG: intervention group.

^h^CG: control group.

^i^PCL-5: Posttraumatic Stress Disorder Checklist–5.

^j^NS: not specified in the study.

^k^SQ: sleep quality.

^l^MADRS-S: Montgomery-Åsberg Depression Rating Scale–Self-rated.

^m^PSQI: Pittsburgh Sleep Quality Index.

^n^FSS: Fatigue Severity Scale.

^o^HADS-D: Hospital Anxiety and Depression Scale–Depression.

^p^QIDS: Quick Inventory of Depressive Symptomatology.

^q^CES-D: Center for Epidemiologic Studies Depression Scale.

^r^SCI: Sleep Condition Indicator.

^s^DBAS: Dysfunctional Beliefs and Attitudes about Sleep.

^t^MFI: Multidimensional Fatigue Inventory.

^u^HADS-A: Hospital Anxiety and Depression Scale–Anxiety.

^v^SF-36: 36-item Short Form Survey.

^w^HADS: Hospital Anxiety and Depression Scale.

^x^ADS-K: Allgemeine Depressions-Skala-Kurzform.

^y^QoL: quality of life.

^z^BDI: Beck Depression Inventory.

^aa^BSI-Anxiety: Brief Symptom Inventory–Anxiety.

^ab^BAI: Beck Anxiety Inventory.

^ac^BNSQ: Basic Nordic Sleep Questionnaire.

^ad^BBQ: Brunnsviken Brief Quality of Life Scale.

^ae^K6: Kessler Psychological Distress Scale.

^af^QoL-VAS: Quality of Life-Visual Analog Scale.

^ag^CFQ: 11-item Chalder Fatigue Questionnaire.

### Effects of eHealth-Based Psychosocial Interventions on Primary Outcomes in Comparison With Inactive Controls

**Figure 2:**
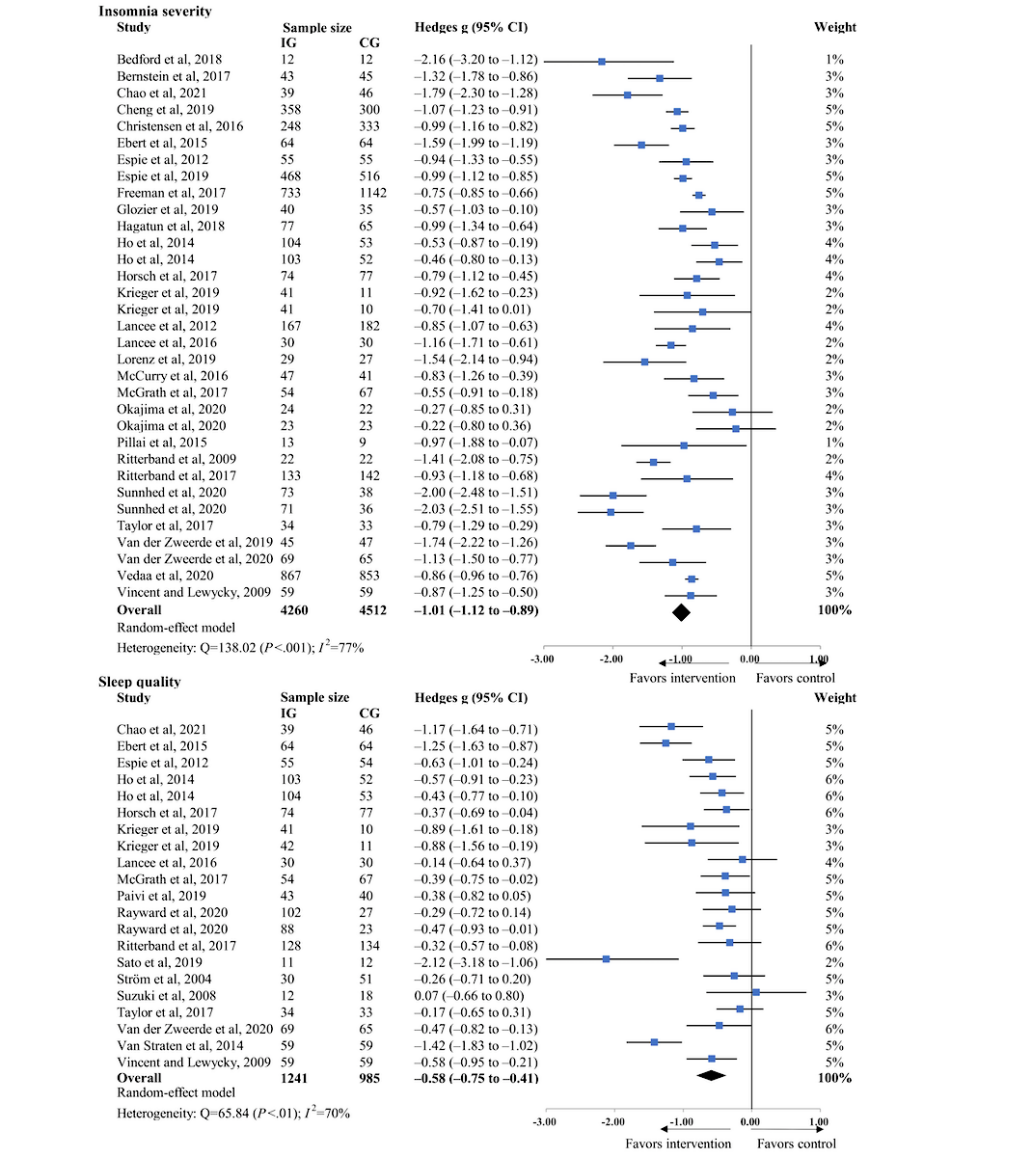
Effects of eHealth-based psychosocial interventions on primary outcomes in comparison with inactive controls [13,35-39,53,54,56-81]. CG: control group; IG: intervention group.

The pooled effect sizes from the study comparing eHealth-based psychosocial interventions with inactive controls on insomnia severity and sleep quality are presented in Figure 2 [13,35-39,53,54,56-81], along with the individual effects of each intervention trial. eHealth-based psychosocial interventions were effective in reducing insomnia severity compared with inactive controls (29/37, 78% of the studies; Hedges *g*=−1.01, 95% CI −1.12 to −0.89; *P*<.001). There was substantial heterogeneity across the studies (*I*^2^=77%; *P*<.001). In total, 49% (18/37) of the studies assessing sleep quality found that eHealth-based psychosocial interventions led to a moderate improvement in sleep quality (Hedges *g*=−0.58, 95% CI −0.75 to −0.41; *P*<.001). We found substantial heterogeneity across the studies (*I*^2^=69.6%; *P*<.001). However, both the visual inspection of the funnel plot and the Egger regression test revealed no clear evidence of potential publication bias (Multimedia Appendix 3). The influence analysis identified no outlier studies by removing each study to recalculate the pooled effect size (see the results of the influence analysis in Multimedia Appendix 4 [12,13,35-39,53-81]).

### Effects of eHealth-Based Psychosocial Interventions on Primary Outcomes in Comparison With In-Person CBT

**Figure 3:**
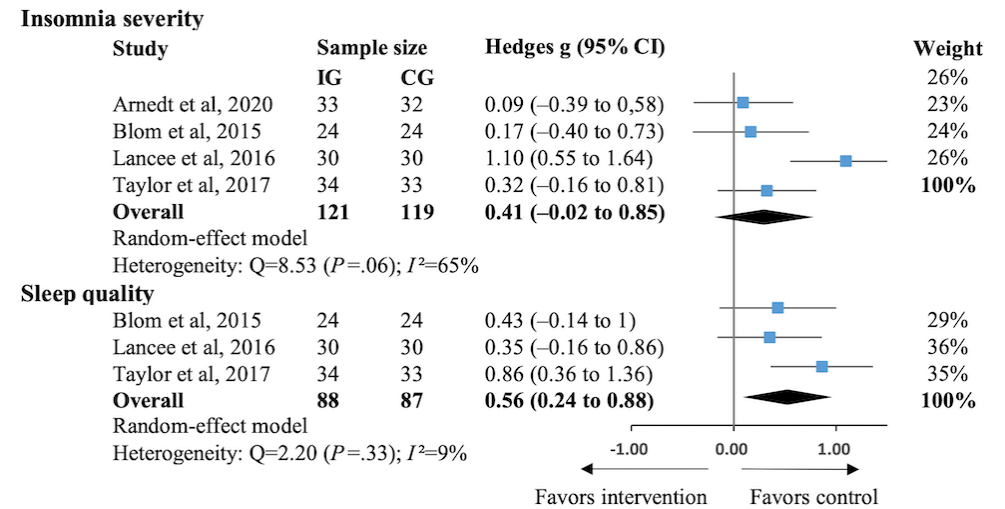
Effects of eHealth-based psychosocial interventions on primary outcomes in comparison with in-person cognitive behavioral therapy (CBT) [26,55,67,78]. CG: control group; IG: intervention group.

The results of the eHealth-based psychosocial interventions versus in-person CBT are shown in Figure 3 [26,55,67,78]. We found that in-person CBT showed greater improvement in insomnia severity (4/37, 11% of the studies; Hedges *g*=0.41, 95% CI −0.02 to 0.85; *P*=.06; *I*^2^=65%) compared with eCBT; however, the assumption of noninferiority of eHealth interventions compared with in-person CBT was not rejected (*P*=.06). In terms of improving sleep quality, in-person interventions had a significantly superior performance (3/37, 8% of the studies; Hedges *g*=0.56, 95% CI 0.24-0.88; *P*<.001; *I*^2^=9%). Heterogeneity was low to moderate across the studies.

### Exploratory Subgroup Analyses and Metaregression Analyses

The results of the exploratory subgroup analyses conducted using only the studies comparing eHealth-based psychosocial interventions with inactive controls are shown in Table 4. eHealth-based psychosocial interventions had a significant effect on alleviating insomnia severity in clinically diagnosed patients with insomnia (Hedges *g*=−1.15, 95% CI −1.32 to −0.97; *P*<.001; *I*^2^=72%) and subclinical samples (Hedges *g*=−0.86, 95% CI −1 to −0.72; *P*<.001; *I*^2^=76%). There was a significant difference between populations (*Q*=6.83; *P*=.01). With regard to sleep quality, eHealth interventions had a significant effect on clinical patients (Hedges *g*=−0.68, 95% CI −0.94 to −0.42; *P*<.001; *I*^2^=76%) and subclinical samples (Hedges *g*=−0.49, 95% CI −0.70 to −0.28; *P*<.001; *I*^2^=60%); however, there was no statistically significant between-subgroup difference (*Q*=1.24; *P*=.27).

**Table 4:** Exploratory subgroup analyses of effects on the primary outcomes (N=37).

Subgroup				Studies, n (%)		Sample size (IG^a^/CG^b^), n		Meta-analysis				Heterogeneity							Between-group test		
								Hedges *g* (95% CI)		*P* value		*Q*		*P* value		*I*^2^ (%)		*Q*			*P* value
**Insomnia severity**																					
	**Population**																				
		Clinical sample	16 (43)		1367/1185		−1.15 (−1.32 to −0.97)		*<.001* ^c^		59.67		<.001		72		—^d^			—	
		Subclinical sample	13 (35)		2893/3327		−0.86 (−1.00 to −0.72)		*<.001*		58.39		<.001		76		6.83			*.01*	
	**CBT^e^-based intervention**																				
		eCBT^f^	26 (70)		4016/4371		−0.96 (−1.06 to −0.86)		*<.001*		81.51		<.001		68		—			—	
		Non-CBT	4 (11)		244/141		−1.21 (−1.97 to −0.46)		*<.001*		47.57		<.001		89		0.41			.52	
	**Delivery mode of eHealth**																				
		Phone-delivered	4 (11)		207/209		−0.79 (−1.28 to −0.30)		*.002*		21.55		<.001		81		—			—	
		Computer-assisted	10 (27)		562/517		−1.08 (−1.34 to −0.82)		*<.001*		30.20		<.001		70		—			—	
		Multiple devices	16 (43)		3491/3786		−1.02 (−1.15 to −0.89)		*<.001*		85.01		<.001		80		1.03			.60	
	**Guidance modality**																				
		Guided by trained therapist	13 (35)		916/748		−1.19 (−1.45 to −0.92)		*<.001*		76.36		<.001		82		—			—	
		Guided by animated therapist	8 (22)		1722/2128		−0.96 (−1.15 to −0.78)		*<.001*		27.94		<.001		75		—			—	
		No guidance	10 (27)		1622/1636		−0.83 (−0.96 to −0.70)		*<.001*		17.51		.04		49		6.05			*.05*	
	**Feedback**																				
		Tailored feedback	25 (68)		3944/4246		−1.05 (−1.17 to −0.93)		*<.001*		121.70		<.001		79		—			—	
		No tailored feedback	6 (16)		316/266		−0.76 (−1.05 to −0.47)		*<.001*		12.57		.03		60		3.27			.07	
	**Reminder**																				
		Reminder or encouragement	26 (70)		4126/4381		−0.99 (−1.11 to −0.88)		*<.001*		115.46		<.001		77		—			—	
		No reminder or encouragement	5 (14)		134/131		−1.15 (−1.80 to −0.49)		*<.001*		21.34		<.001		81		0.20			.65	
**Sleep quality**																					
	**Population**																				
		Clinical sample	11 (30)		597/564		−0.68 (−0.94 to −0.42)		*<.001*		44.93		<.001		76		—			—	
		Subclinical sample	7 (19)		644/421		−0.49 (−0.70 to −0.28)		*<.001*		20.00		<.001		60		1.24			.27	
	**Therapeutic approach**																				
		eCBT	16 (43)		967/885		−0.61 (−0.80 to −0.41)		*<.001*		62.74		<.001		74		—			—	
		Non-CBT	3 (8)		274/100		−0.43 (−0.67 to −0.19)		*<.001*		2.10		.55		0		1.20			.27	
	**Delivery mode of eHealth**																				
		Phone-delivered	3 (8)		303/173		−0.56 (−0.93 to −0.19)		*.003*		9.71		.02		69		—			—	
		Computer-assisted	9 (24)		439/402		−0.58 (−0.88 to −0.28)		*<.001*		34.07		<.001		77		—			—	
		Mixed methods	7 (19)		499/410		−0.61 (−0.87 to −0.34)		*<.001*		21.95		.003		68		0.05			.98	
	**Guidance modality**																				
		Guided by trained therapist	11 (30)		737/509		−0.75 (−1.00 to −0.50)		*<.001*		47.36		<.001		75		—			—	
		Guided by animated therapist	2 (5)		109/121		−0.50 (−0.76 to −0.24)		*<.001*		0.81		.37		0		—			—	
		No guidance	5 (14)		395/355		−0.33 (−0.48 to −0.18)		*<.001*		2.02		.85		0		8.10			*.02*	
	**Feedback**																				
		Tailored feedback	15 (41)		961/756		−0.62 (−0.84 to −0.41)		*<.001*		63.12		<.001		75		—			—	
		No tailored feedback	4 (11)		280/229		−0.44 (−0.62 to −0.26)		*<.001*		0.85		.84		0		1.70			.19	
	**Reminder**																				
		Reminder or encouragement	17 (46)		1098/886		−0.56 (−0.74 to −0.38)		*<.001*		58.23		<.001		69		—			—	
		No reminder or encouragement	2 (5)		143/99		−0.79 (−1.51 to −0.06)		*.03*		6.48		.001		85		0.35			.55	

^a^IG: intervention group.

^b^CG: control group.

^c^Italicized *P* values are significant.

^d^Differences between subgroups are shown in the next row.

^e^CBT: cognitive behavioral therapy.

^f^eCBT: eHealth-based cognitive behavioral therapy.

Exploratory subgroup analyses pivoting on intervention features showed that “guidance by trained therapists” and “tailored feedback” were moderators that caused significant differences in effects. Specifically, eHealth-based psychosocial interventions providing “guidance by trained therapists” were more effective in reducing insomnia severity (Hedges *g*=−1.19, 95% CI −1.45 to −0.92; *P*<.001; *I*^2^=82%) than those guided by animated therapists (Hedges *g*=−0.96, 95% CI −1.15 to −0.78; *P*<.001; *I*^2^=75%) and those without guidance (Hedges *g*=−0.83, 95% CI −0.96 to −0.70; *P*<.001; *I*^2^=49%). The difference between the subgroups was approximately significant (*Q*=6.05; *P*=.05). Regarding sleep quality, eHealth interventions that provided user guidance by trained therapists had greater absolute effect sizes (Hedges *g*=−0.75, 95% CI −1.00 to −0.50; *P*<.001; *I*^2^=75%) than those that used animated therapists to guide participants (Hedges *g*=−0.50, 95% CI −0.76 to −0.24; *P*<.001; *I*^2^=0) and those without any guidance (Hedges *g*=−0.33, 95% CI −0.48 to −0.18; *P*<.001; *I*^2^=0). The difference between subgroups was significant (*Q*=8.10; *P*=.02). In addition, eHealth interventions providing “tailored feedback” were more effective in reducing insomnia severity (Hedges *g*=−1.05, 95% CI −1.17 to −0.93; *P*<.001; *I*^2^=79%) than those without tailored feedback (Hedges *g*=−0.76, 95% CI −1.05 to −0.47; *P*<.001; *I*^2^=60%). The difference between the subgroups was the marginal significance level (*Q*=3.27; *P*=.07). However, there were no statistically significant associations between therapeutic approach, eHealth delivery mode, or provision of reminders and effect sizes.

The results of the metaregression analyses are presented in Table 5. The baseline insomnia severity and intervention duration (in weeks and sessions) had moderating effects on the study effects on insomnia severity. Higher baseline insomnia severity was associated with larger effect sizes (*b*=−0.11; *P*=.004. In addition, a longer intervention duration was associated with larger effect sizes (in weeks: *b*=−0.09 and *P*=.01 in sessions: *b*=−0.20 and *P*=.03). See the plots of the metaregression analyses in Multimedia Appendix 5. However, no statistically significant associations were observed among sleep medication, number of intervention components, number of eHealth functions, and the effects on insomnia severity or between these moderators and the effects on sleep quality.

**Table 5:** Metaregression analyses of effects on the primary outcomes (N=37).

Moderator			Studies, n (%)		*b* (95% CI)		*P* value
**Baseline insomnia severity**							
	Insomnia severity	28 (76)		−0.11 (−0.18 to −0.04)		*.004* ^a^	
	Sleep quality	14 (38)		0.03 (−0.05 to 0.11)		.44	
**Sleep medication**							
	Insomnia severity	17 (46)		−1.28 (−3.04 to 0.48)		.14	
	Sleep quality	11 (30)		0.18 (−0.15 to 0.51)		.26	
**Intervention duration (weeks)**							
	Insomnia severity	32 (86)		−0.09 (−0.16 to −0.02)		*.01*	
	Sleep quality	21 (57)		−0.003 (−0.09 to 0.09)		.94	
**Intervention duration (sessions)**							
	Insomnia severity	25 (68)		−0.20 (−0.38 to −0.03)		*.03*	
	Sleep quality	15 (41)		−0.03 (−0.32 to 0.26)		.81	
**Number of intervention components**							
	Insomnia severity	30 (81)		−0.04 (−0.13 to 0.06)		.46	
	Sleep quality	21 (57)		−0.04 (−0.17 to 0.09)		.57	
**Number of eHealth functions**							
	Insomnia severity	33 (89)		−0.02 (−0.19 to 0.15)		.81	
	Sleep quality	21 (57)		−0.14 (−0.33 to 0.05)		.14	

^a^Italicized *P* values are significant.

### Effects of eHealth-Based Psychosocial Interventions on Secondary Outcomes

**Figure 4:**
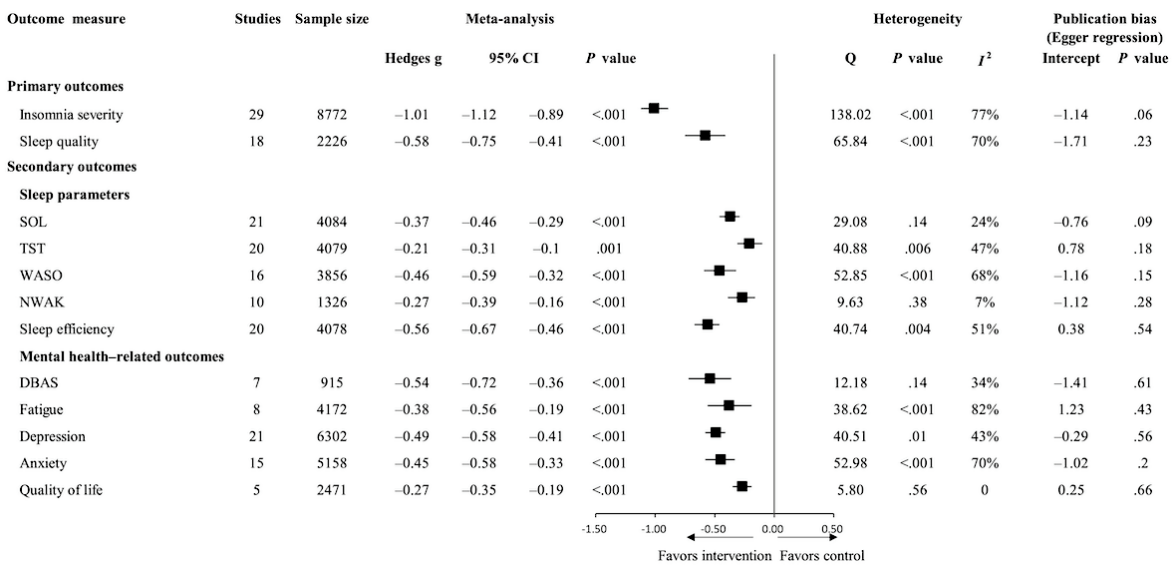
Effects of eHealth-based psychosocial interventions on outcome measures. DBAS: Dysfunctional Beliefs and Attitudes about Sleep; NWAK: number of nocturnal awakenings; SOL: sleep onset latency; TST: total sleep time; WASO: wake after sleep onset.

All the pooled effect sizes related to sleep parameters and mental health–related outcomes remained statistically significant, as shown in Figure 4, with negative Hedges *g* values indicating a direction in favor of eHealth-based psychosocial interventions. Analyses indicated that, compared with changes in controls, eHealth-based psychosocial interventions significantly increased the TST (Hedges *g*=−0.21, 95% CI −0.31 to −0.10; *P*=.001) and sleep efficiency (Hedges *g*=−0.56, 95% CI −0.67 to −0.46; *P*<.001) of participants, shortened the SOL (Hedges *g*=−0.37, 95% CI −0.46 to −0.29; *P*<.001) and WASO (Hedges *g*=−0.46, 95% CI −0.59 to −0.32; *P*<.001), and reduced the NWAK (Hedges *g*=−0.27, 95% CI −0.39 to −0.16; *P*<.001).

Furthermore, the eHealth-based psychosocial interventions significantly reduced participants’ maladaptive beliefs about sleep (Hedges *g*=−0.54, 95% CI −0.72 to −0.36; *P*<.001), fatigue (Hedges *g*=−0.38, 95% CI −0.56 to −0.19; *P*<.001), depression symptoms (Hedges *g*=−0.49, 95% CI −0.58 to −0.41; *P*<.001), and anxiety symptoms (Hedges *g*=−0.45, 95% CI −0.58 to −0.33; *P*<.001) and improved quality of life (Hedges *g*=−0.27, 95% CI −0.35 to −0.19; *P*<.001) compared with the controls. The analyses revealed low to substantial heterogeneity (*I*^2^=0%-77%). The Egger test of publication bias did not reach statistical significance for the secondary outcomes.

### Quality Assessment

**Figure 5:**
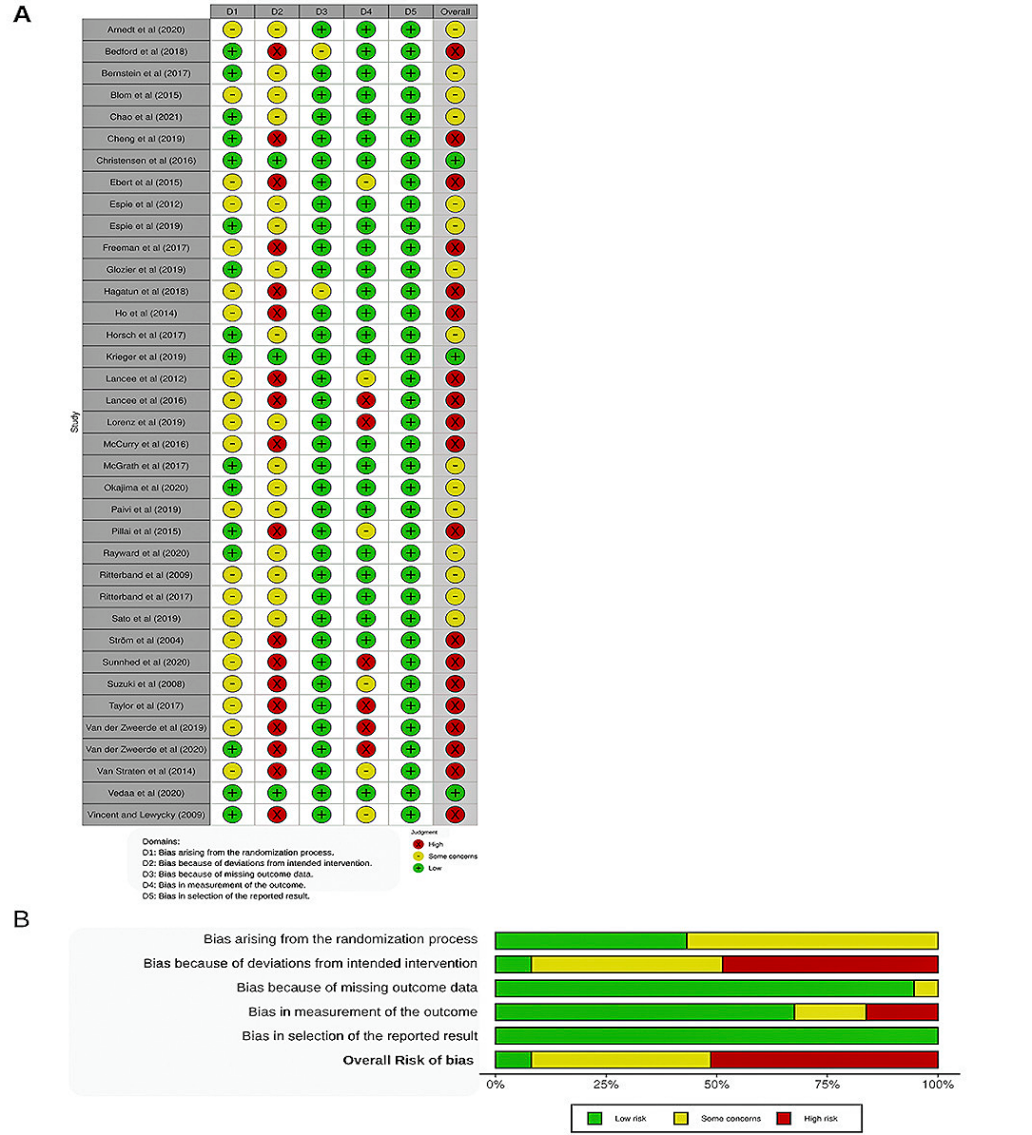
Risk-of-bias assessment of the included studies [12,13,26,35-39,53-81].

The results from the Cochrane risk-of-bias tool for randomized trials assessment are shown in Figure 5 [12,13,26,35-39,53-81]. Of the studies, 54% (20/37) were rated as having a high overall risk of bias, 41% (15/37) were rated as having some concerns regarding the overall bias, and 5% (2/37) had a low overall risk of bias. The most frequent risk factor identified was *deviations from intended interventions*, which was most often owing to inadequate blinding of participants or caregivers, adherence problems, or lack of appropriate analysis; 49% (18/37) of the studies were evaluated as having a high risk of bias in this domain, with 43% (16/37) of the studies evaluated as having some concerns. Owing to a lack of information on concealment of the allocation sequence, 57% (21/37) of the included studies had some concerns regarding the *randomization process*. A total of 95% (35/37) and 68% (25/37) of the studies were evaluated as having a low risk of bias for *missing outcome data* and *measurement of the outcome*, respectively. None of the included studies was evaluated as having a risk of bias regarding *selectively reported results*.

## Discussion

### Principal Findings

This systematic review identified 37 RCTs that reported data on 13,227 individuals from 12 countries. eHealth-based psychosocial interventions were delivered via a website, computer, smartphone, telephone, or mixed mode, in most cases (32/37, 86%) based on CBT-I. Questionnaires and sleep diaries were used to evaluate the effectiveness. Our findings are outlined in Textbox 2.

**Summary of findings.**

- We found that eHealth-based psychosocial interventions yielded a large reduction in insomnia severity (Hedges *g*=−1.01) and a moderate improvement in sleep quality (Hedges *g*=−0.58) as compared with inactive controls. Despite the heterogeneity between studies on primary outcomes, no outlier studies were identified through influence analysis, funnel plots, and Egger tests, indicating no significant publication bias.
- There was no significant difference in the reduction of insomnia severity when comparing eHealth interventions and in-person cognitive behavioral therapy (CBT). However, in-person CBT was shown to be more effective in improving sleep quality.
- Clinical samples (Hedges *g*=−1.15) benefited significantly more from eHealth interventions in reducing insomnia severity than subclinical samples (Hedges *g*=−0.86).
- Guidance from trained therapists and tailored feedback were associated with larger treatment effects on insomnia symptoms. All subgroup analyses on primary outcomes favored eHealth interventions and indicated at least moderate effect sizes (Hedges *g*=−0.33 to −1.21).
- Higher baseline insomnia severity and longer intervention duration were associated with a larger reduction in insomnia severity.
- With respect to secondary outcomes, eHealth interventions for insomnia had significantly small to moderate positive effects (Hedges *g*=−0.21 to −0.56) on sleep parameters and mental health–related outcomes.

### Comparison With Prior Work

Our main findings suggest that eHealth-based psychosocial interventions had a positive effect on the management of insomnia symptoms. Similar results were reported in an earlier meta-analysis published in 2016, which included 11 RCTs of digital CBT-I in the adult population (effect sizes: −0.89 and −0.49) [31]. A recent meta-analysis of digital CBT-I in adolescents or college students combined the effect sizes of 4 RCTs and reported slightly larger effects on sleep quality (−0.58) [85]. These results suggest that eHealth-based psychosocial interventions are a promising solution for managing insomnia.

eHealth-based psychosocial interventions had substantially larger effects on improving insomnia severity and sleep quality than inactive controls. However, we found no significant difference between eCBT and in-person CBT in improving insomnia severity despite a significant difference in enhanced sleep quality. All 11% (4/37) of the studies with an in-person comparison group concluded that eCBT offered a potentially cost-effective alternative with low-cost labor, with approximately 38% of the time investment associated with face-to-face treatment [26,55,67,78]. Intensive therapist support, high concentration of attention, prompt feedback, and substantial time commitment in face-to-face treatment may explain the differential effects in trials [26,67]. Our findings support the notion that eHealth-based treatments could be a potentially cost-effective alternative to in-person treatment. This finding is in line with previous research showing that digital CBT-I has similar efficacy to face-to-face CBT [32,86]. However, it is impossible to draw definitive conclusions from only 4 original trials. Additional noninferiority trials comparing eHealth-based psychosocial interventions with in-person treatment for insomnia are warranted, and the specific psychotherapy elements used should be dissected, quantified, and evaluated in future studies [31,87].

In addition, our review showed a larger improvement in insomnia severity in the clinical samples and samples with higher baseline insomnia severity. Possible explanations for this include clinical samples with more severe insomnia symptoms having more space for improvement. In addition, user requirements and motivation may influence attention engagement and, thus, treatment effectiveness. The observed effects suggest that eHealth-based psychosocial interventions for insomnia could be applicable to a broad range of populations and are well suited to be integrated into a stepped-care approach [88,89].

With regard to eHealth intervention features, our results imply that professional guidance and tailored feedback are associated with greater effect sizes. Thus, this could be crucial in facilitating the effectiveness of eHealth-based psychosocial interventions as the involvement of trained therapist support and individualized advice seem to promote effectiveness. This further supports the view that blended interventions integrating therapeutic support in face-to-face treatment with the cost-effectiveness of eHealth could be a way to increase treatment effectiveness while saving time and reducing costs [90]. Contrary to expectations, eCBT and other psychosocial interventions were found to be roughly equivalent in effectiveness, although caution is needed as only 4 non-CBT eHealth studies were included in this review. Alternative therapeutic techniques such as acceptance and commitment therapy, problem-solving therapy, psychodynamic therapy, mindfulness therapy, and interpersonal psychotherapy are also worth exploring in conjunction with eHealth to promote sleep and well-being. Considering the structured nature of CBT, it might not be beneficial for people with complex needs, and it fails to address deeper causes or the possible underlying causes of mental illness, such as childhood experience, family history, or relations [91]. In addition, the typical treatment period for CBT is 6 to 20 weeks, which, to some extent, requires people to adhere to a long period. Indeed, a more thorough investigation of the effectiveness of different therapeutic techniques implemented in eHealth for the treatment of insomnia is necessary. Furthermore, a longer intervention duration was found to be associated with a larger reduction in insomnia severity; similar results were reported in a previous meta-analysis on CBT-I [31]. Finally, no significant effects on insomnia were found for moderators such as delivery mode, reminder settings, sleep medication use, number of intervention components, and eHealth functions. Additional studies are needed to determine optimal intervention characteristics, including the number of treatment sessions and intervention components.

We further found that eHealth-based psychosocial interventions effectively improved sleep efficiency and TST and reduced SOL, WASO, and NWAK. These findings are in line with those of previous studies [31,86]. There is also evidence supporting a mixed effect on mental health–related outcomes, adjusting people’s maladaptive beliefs about sleep and alleviating fatigue, anxiety, and depression symptoms. Thus, eHealth-based psychosocial interventions for insomnia could promote mental health and prevent the exacerbation of comorbid medical and psychiatric conditions. Previous studies have demonstrated the effects of eHealth interventions for cancer survivors on improving sleep and reducing fear of recurrence, depression, and anxiety [92,93]. These low-cost and convenient insomnia treatments can be widely disseminated, along with support for eHealth-illiterate populations, among people with mental or physical disorders accompanied by insomnia [94].

This meta-analysis focused on a wide range of sleep and mental health outcome measures to assess the impact of the intervention in a holistic manner. Our findings highlight several directions for future research. Given the importance of user engagement and therapist support for treatment effectiveness and adherence, future research could pay more attention to increasing user engagement and interaction in the design of eHealth-based psychosocial interventions for insomnia. This could be achieved by developing blended, appealing, and adaptive interventions as well as making eHealth interventions accessible to those with a lower eHealth literacy [22]. Furthermore, research is needed to directly compare eHealth-based psychosocial interventions in different delivery modes in noninferiority trials while assessing the cost-effectiveness, treatment credibility, satisfaction, and therapeutic alliance. This would help tremendously in the optimization of eHealth-based psychosocial interventions. In addition, given that most trials to date have been implemented in high-income countries with little cultural diversity, eHealth-based psychosocial interventions for insomnia should also be investigated in more low- and middle-income countries to increase the accessibility of eHealth.

### Limitations

Some limitations should be noted. First, this review only focused on adults with insomnia; further research is needed to evaluate the effects of eHealth-based psychosocial interventions in specific populations, including children, adolescents or employees of specific sectors. Second, although the random-effects model aimed to account for between-study heterogeneity statistically, our analyses still indicated significant between-study differences. Variability in the control condition was particularly identified as a source of heterogeneity. However, the heterogeneity in this study might be multivariate, which may be caused by different outcome measures or confounding bias. As not all studies used the same outcomes, the pooled effects for insomnia severity and sleep quality and the secondary outcomes were based on different numbers of studies and, to some extent, using different outcomes. The difference in outcome measures might more or less influence the treatment effects. Third, our meta-analyses focused on the immediate intervention effects. Owing to a certain level of dropout, few studies assessed insomnia symptoms for >6 months, and the included studies set different follow-up times. Future studies could include long-term follow-ups at standardized lags to observe the prolonged effects on sleep. Fourth, this review depicted participants’ completion of various questionnaires and assessments. However, because of the limited number of studies that reported the same outcome measure on treatment adherence, we failed to summarize how participants implemented treatment recommendations and how therapists followed treatment protocols, which are important factors in treatment adherence [95]. The impact of treatment adherence on eHealth-based psychosocial interventions is worth investigating, and future RCTs should use standardized methods to comprehensively assess treatment adherence and its relationship with treatment effects.

### Conclusions

In conclusion, this review provides an up-to-date and comprehensive overview and quantitative integration of current research on the effectiveness of eHealth-based psychosocial interventions for insomnia. eHealth-based psychosocial interventions have the potential to reduce both insomnia symptoms and other mental health–related outcomes. Our findings suggest that, as a less costly intervention, eHealth-based psychosocial interventions should be disseminated widely and integrated into a stepped-care model. Professional guidance and tailored feedback should accompany eHealth interventions to improve effectiveness. Blended care integrating face-to-face care with eHealth may further improve effectiveness and benefit a more diverse population with insomnia complaints. Further investigations of intervention components and blended interventions are needed to better understand the effectiveness of different intervention components, especially as pertaining to people of low socioeconomic status or low eHealth literacy.

## Appendix

Multimedia Appendix 1 Search terms.

Multimedia Appendix 2 Studies with specific characteristics.

Multimedia Appendix 3 Funnel plots of studies included in the meta-analyses.

Multimedia Appendix 4 Influence of individual studies on the effect size.

Multimedia Appendix 5 Plots of meta-regressions.

## Abbreviations

CBT: cognitive behavioral therapy
CBT-I: cognitive behavioral therapy for insomnia
eCBT: eHealth-based cognitive behavioral therapy
NWAK: number of nocturnal awakenings
PRISMA: Preferred Reporting Items for Systematic Reviews and Meta-Analyses
RCT: randomized controlled trial
SOL: sleep onset latency
TST: total sleep time
WASO: wake after sleep onset
