# Supplementary material for: eHealth-Based Psychosocial Interventions for Adults With Insomnia: Systematic Review and Meta-analysis of Randomized Controlled Trials
Source: J Med Internet Res. 2023 Mar 14;25:e39250. doi: 10.2196/39250 (PMC10131777; doi:10.2196/39250)
Supplement: Multimedia Appendix 1 [file jmir_v25i1e39250_app1.docx]

**Multimedia Appendix 1: Search terms.**

**Search terms**

A

(‘Sleep Initiation and Maintenance Disorders’[Mesh] OR ‘insomni*’[tiab] OR ‘sleepless*’[tiab] OR ‘sleep problem*’[tiab] OR ‘sleep disorder*’[tiab] OR ‘sleep disturbance*’[tiab] OR ‘sleep dysfunction’[tiab] OR ‘sleeping problem*’[tiab] OR ‘sleeping disorder*’[tiab] OR ‘sleeping disturbance*’[tiab] OR ‘sleeping dysfunction’[tiab])

B

(‘Internet[Mesh]’ OR ‘Telemedicine’[Mesh] OR ‘telemedicine’[tiab] OR ‘teleconference’[tiab] OR ‘telecommunication’[tiab] OR ‘telehealth’[tiab] OR ‘telecare’[tiab] OR ‘electronic health’[tiab] OR ‘mobile health’[tiab] OR ‘mHealth’[tiab] OR ‘eHealth’[tiab] OR ‘telephone’[tiab] OR ‘mobile phone’[tiab] OR ‘cell phone’[tiab] OR ‘cellular phone’[tiab] OR ‘smartphone’[tiab] OR ‘smart phone’[tiab] OR ‘mobile technology’ OR ‘wireless’[tiab] OR ‘internet’[tiab] OR ‘internet-based’[tiab] OR ‘computer*’[tiab] OR ‘computer-assisted instruction’[tiab] OR ‘multimedia’[tiab] OR ‘email*’[tiab] OR ‘e-mail’[tiab] OR ‘web’[tiab] OR ‘web based’[tiab] OR ‘web-based’[tiab] OR ‘online’[tiab] OR ‘technolog*’[tiab] OR ‘digital’[tiab] OR ‘text messag*’[tiab] OR ‘SMS’[tiab] OR ‘remote consultation’[tiab] OR ‘telemonitoring’[tiab] OR ‘iphone’[tiab] OR ‘i-phone’[tiab] OR ‘social media’[tiab] OR ‘virtual community’[tiab] OR ‘medical informatics’[tiab])

C

(‘Psychosocial Intervention’[Mesh] OR ‘Psychosocial Support Systems’[Mesh] OR ‘Patient Education as Topic’[Mesh] OR ‘intervention’[tiab] OR ‘education’[tiab] OR ‘Health Education’[Mesh] OR ‘information’[tiab] OR ‘train*’[tiab] OR ‘coach’[tiab] OR ‘skills’[tiab] OR ‘program*’[tiab] OR ‘counseling’[tiab] OR ‘mentor’[tiab] OR ‘care’[tiab] OR ‘support’[tiab] OR ‘health promotion’[tiab] OR ‘lifestyle’[tiab] OR ‘therapy’[tiab] OR ‘manage*’[tiab] OR ‘psycho*’[tiab] OR ‘treat*’[tiab] OR ‘relax*’[tiab] OR ‘rehabilitation’[tiab] OR ‘monitor’[tiab] OR ‘record*’[tiab] OR ‘CBT’[tiab] OR ‘Cognitive Behavioral Therapy’[Mesh] OR ‘Cognitive Behavioral Therapy’[tiab] OR ‘Cognitive Behavioural Therapy’[tiab] OR ‘Cognitive Behavior Therapy’[tiab] OR ‘Cognitive Behaviour Therapy’[tiab] OR ‘CBT-i’[tiab] OR ‘CBTI’[tiab] OR ‘meditation’[tiab] OR ‘mindful*’[tiab] OR ‘stimulation control’[tiab] OR ‘sleep restriction’[tiab] OR ‘sleep hygiene’[tiab] OR ‘behavior*’[tiab] OR ‘behaviour*’[tiab] OR ‘imag*’[tiab] OR ‘suggestion’[tiab] OR ‘crisis intervention’[tiab] OR ‘dream analysis’[tiab] OR ‘emotional freedom’[tiab] OR ‘free association’[tiab] OR ‘hypno*’[tiab] OR ‘guide*’[tiab] OR ‘self*’[tiab] OR ‘individual’[tiab])

| **Database** | **Search strategy** | **Result (Number of hits)** |
| --- | --- | --- |
| Pubmed | A AND B AND C | 3741 |
| EMBASE | A AND B AND C | 3655 |
| Web of Science | A AND B AND C | 4279 |
| PsycINFO | A AND B AND C | 6697 |
| Cochrane | A AND B AND C | 1605 |
