# Supplementary material for: eHealth-Based Psychosocial Interventions for Adults With Insomnia: Systematic Review and Meta-analysis of Randomized Controlled Trials
Source: J Med Internet Res. 2023 Mar 14;25:e39250. doi: 10.2196/39250 (PMC10131777; doi:10.2196/39250)
Supplement: Multimedia Appendix 2 [file jmir_v25i1e39250_app2.docx]

**Multimedia Appendix 2:**

# **Intervention with specific characteristics.**

| Characteristics | | Study |
| --- | --- | --- |
| **Comparison type** | |  |
|  | eHealth interventions vs. in-person CBT (11%, n*=*4) | Arnedt et al (2020), Blom et al (2015), Lancee et al (2016), Taylor et al (2017) |
|  | eHealth interventions vs. inactive controls (95%, n*=*35) | Bedford et al (2018), Bernstein et al (2017), Chao et al (2021), Cheng et al (2019), Christensen et al (2016), Ebert et al (2015), Espie et al (2012), Espie et al (2019), Freeman et al (2017), Glozier et al (2019), Hagatun et al (2018), Ho et al (2014), Horsch et al (2017), Krieger et al (2019), Lancee et al (2012), Lorenz et al (2019), McCurry et al (2016), McGrath et al (2017), Okajima et al (2020), Paivi et al (2019), Pillai et al (2015), Rayward et al (2020), Ritterband et al (2009), Ritterband et al (2017), Sato et al (2019), Ström et al (2004), Sunnhed et al (2020), Suzuki et al (2008), van der Zweerde et al (2019), van der Zweerde et al (2020), van Straten et al (2014), Vedaa et al (2020), Vincent et al (2009), Lancee et al (2016), Taylor et al (2017) |
| **Population** | |  |
|  | Clinical sample (54%, n=20) | Arnedt et al (2020), Lancee et al (2016), Taylor et al (2017); Lancee et al (2012),  Chao et al (2021), Cheng et al (2019), Espie et al (2012), Glozier et al (2019), Hagatun et al (2018), Krieger et al (2019), Pillai et al (2015), Ritterband et al (2009), Ritterband et al (2017), Sato et al (2019), Ström et al (2004), Sunnhed et al (2020), van der Zweerde et al (2019), van der Zweerde et al (2020), van Straten et al (2014), Vincent et al (2009) |
|  | Subclinical sample (46%, n*=*17) | Blom et al (2015);  Bedford et al (2018), Bernstein et al (2017), Christensen et al (2016), Ebert et al (2015), Espie et al (2019), Freeman et al (2017), Ho et al (2014), Horsch et al (2017), Lorenz et al (2019), McCurry et al (2016), McGrath et al (2017), Okajima et al (2020), Paivi et al (2019), Rayward et al (2020), Suzuki et al (2008), Vedaa et al (2020) |
| **Therapeutic approach** | |  |
|  | eCBT (86%, n*=*32) | Arnedt et al (2020), Blom et al (2015), Lancee et al (2016), Taylor et al (2017);  Bernstein et al (2017), Chao et al (2021), Cheng et al (2019), Christensen et al (2016), Ebert et al (2015), Espie et al (2012), Espie et al (2019), Freeman et al (2017), Glozier et al (2019), Hagatun et al (2018), Ho et al (2014), Horsch et al (2017), Krieger et al (2019), Lancee et al (2012), Lorenz et al (2019), McCurry et al (2016), McGrath et al (2017), Pillai et al (2015), Ritterband et al (2009), Ritterband et al (2017), Sato et al (2019), Ström et al (2004), Suzuki et al (2008), van der Zweerde et al (2019), van der Zweerde et al (2020), van Straten et al (2014), Vedaa et al (2020), Vincent et al (2009) |
|  | Non-CBT (16%, n*=*6) | Bedford et al (2018), Krieger et al (2019), Okajima et al (2020), Paivi et al (2019), Rayward et al (2020), Sunnhed et al (2020) |
| **Delivery mode** | |  |
|  | Phone-delivered (14%, n=5) | Chao et al (2021), Horsch et al (2017), McCurry et al (2016), Okajima et al (2020), Rayward et al (2020) |
|  | Computer-assisted (35%, n=13) | Arnedt et al (2020); Lancee et al (2016);  Bedford et al (2018), Ho et al (2014), Lancee et al (2012), Lorenz et al (2019), Paivi et al (2019), Sato et al (2019), Ström et al (2004), van der Zweerde et al (2019), van der Zweerde et al (2020), van Straten et al (2014), Vincent et al (2009) |
|  | Mixed-mode (54%, n=20) | Blom et al (2015), Taylor et al (2017);  Bernstein et al (2017), Cheng et al (2019), Christensen et al (2016), Ebert et al (2015), Espie et al (2012), Espie et al (2019), Freeman et al (2017), Glozier et al (2019), Hagatun et al (2018), Ho et al (2014), Krieger et al (2019), McGrath et al (2017), Pillai et al (2015), Ritterband et al (2009), Ritterband et al (2017), Sunnhed et al (2020), Suzuki et al (2008), Vedaa et al (2020) |
| **Guidance modality** | |  |
|  | Guided by trained therapist (46%, n=17) | Arnedt et al (2020), Blom et al (2015), Lancee et al (2016);  Chao et al (2021), Ebert et al (2015), Ho et al (2014), Krieger et al (2019), Lorenz et al (2019), McCurry et al (2016), Okajima et al (2020), Rayward et al (2020), Sato et al (2019), Ström et al (2004), Sunnhed et al (2020), van der Zweerde et al (2019), van der Zweerde et al (2020), van Straten et al (2014) |
|  | Guided by virtual therapist (19%, n=7) | Bedford et al (2018), Cheng et al (2019), Espie et al (2012), Espie et al (2019), Freeman et al (2017), McGrath et al (2017), Pillai et al (2015), |
|  | No guidance (41%, n=15) | Taylor et al (2017);  Bernstein et al (2017), Christensen et al (2016), Glozier et al (2019), Hagatun et al (2018), Ho et al (2014), Horsch et al (2017), Lancee et al (2012), Okajima et al (2020), Paivi et al (2019), Ritterband et al (2009), Ritterband et al (2017), Suzuki et al (2008), Vedaa et al (2020), Vincent et al (2009) |
| **Feedback** | |  |
|  | Tailored feedback (86%, n=32) | Arnedt et al (2020), Blom et al (2015), Lancee et al (2016), Taylor et al (2017);  Bedford et al (2018), Chao et al (2021), Cheng et al (2019), Christensen et al (2016), Ebert et al (2015), Espie et al (2012), Espie et al (2019), Freeman et al (2017), Glozier et al (2019), Hagatun et al (2018), Ho et al (2014), Krieger et al (2019), Lorenz et al (2019), McCurry et al (2016), McGrath et al (2017), Okajima et al (2020), Pillai et al (2015), Rayward et al (2020), Ritterband et al (2009), Ritterband et al (2017), Sato et al (2019), Ström et al (2004), Sunnhed et al (2020), Suzuki et al (2008), van der Zweerde et al (2019), van der Zweerde et al (2020), van Straten et al (2014), Vedaa et al (2020) |
|  | No tailored feedback (19%, n=7) | Bernstein et al (2017), Ho et al (2014), Horsch et al (2017), Lancee et al (2012), Okajima et al (2020), Paivi et al (2019), Vincent et al (2009) |
| **Reminder** | |  |
|  | Reminder or encouragement (92%, n=34) | Arnedt et al (2020), Blom et al (2015), Lancee et al (2016), Taylor et al (2017);  Bedford et al (2018), Bernstein et al (2017), Cheng et al (2019), Christensen et al (2016), Ebert et al (2015), Espie et al (2012), Espie et al (2019), Freeman et al (2017), Glozier et al (2019), Hagatun et al (2018), Ho et al (2014), Horsch et al (2017), Krieger et al (2019), Lancee et al (2012), McGrath et al (2017), Okajima et al (2020), Paivi et al (2019), Pillai et al (2015), Rayward et al (2020), Ritterband et al (2009), Ritterband et al (2017), Sato et al (2019), Ström et al (2004), Sunnhed et al (2020), Suzuki et al (2008), van der Zweerde et al (2019), van der Zweerde et al (2020), van Straten et al (2014), Vedaa et al (2020), Vincent et al (2009) |
|  | No reminder or encouragement (14%, n=5) | Chao et al (2021), Ho et al (2014), Lorenz et al (2019), McCurry et al (2016), Okajima et al (2020) |

# **Process outcomes - Participant satisfaction and negative effects**

| Study | Participant satisfaction - Questionnaire used^a^ | Satisfied or mostly satisfied | Summary (rated as) | Adverse events reported | Report of specific adverse events |  |
| --- | --- | --- | --- | --- | --- | --- |
| Arnedt et al (2020) | CSQ-8 | - ^b^ | High | No^c^ | No | |
| Bedford et al (2018) | - | - | - | - | - | |
| Blom et al (2015) | CSQ-8 | - | High | 6 participants in ICBT | 1. disappointed about the treatment effect; 2. stressful or increased worry about sleep; 3. increased headache and a minor traffic incident after getting little sleep | |
| Cheng et al (2019) | - | - | - | - | - | |
| Ebert et al (2015) | CSQ-8 | 89.8% | Very high | - | - | |
| Espie et al (2012) | - | - | - | No | No | |
| Espie et al (2019) | - | - | - | Potential adverse effects | Unrelated to the intervention | |
| Freeman et al (2017) | - | - | - | No | No | |
| Glozier et al (2019) | - | - | - | 1 participant | Increased HAM-D score | |
| Hagatun et al (2018) | UQ | 83–84% | - | No | No | |
| Ho et al (2014) | 10-point Likert scale | - | Moderate | - | - | |
| Horsch et al (2017) | - | - | - | - | - | |
| Lancee et al (2016) | CSQ-8 | - | Moderate | No | No | |
| Lancee et al (2012) | Usefulness of the modules | - | very useful | - | - | |
| Lorenz et al (2019) | - | - | - | - | - | |
| McCurry et al (2016) | Patient-reported satisfaction | - | High | - | - | |
| McGrath et al (2017) | - | - | - | - | - | |
| Okajima et al (2020) | - | - | - | - | - | |
| Paivi et al (2019) | 10- point Visual Analogue scale | - | High | - | - | |
| Pillai et al (2015) | - | - | - | - | - | |
| Rayward et al (2020) | - | - | - | - | - | |
| Ritterband et al (2009) | - | - | - | - | - | |
| Ritterband et al (2017) | - | - | - | - | - | |
| Sato et al (2019) | - | - | - | - | - | |
| Ström et al (2004) | - | - | - | - | - | |
| Sunnhed et al (2020) | CSQ-8 | - | High | 29% of the total sample | Low mood; fatigue/exhaustion; extreme sleepiness; reduced motivation/energy; feeling irritable ect. | |
| Suzuki et al (2008) | - | - | - | - | - | |
| Taylor et al (2017) | - | - | - | 7 participants | One patient with an increase in insomnia after primary care physician reduced hypnotic medication; six patients with skin irritation from wearing the Actiwatch. | |
| Van der Zweerde et al (2020) | - | - | - | No | No | |
| Van der Zweerde et al (2019) | 10-point Likert scale | - | High | No | No | |
| Van Straten et al (2014) | 10-point Likert scale | 79.6% | High | - | - | |
| Vincent et al (2009) | - | - | - | - | - | |

^a^ CSQ-8 = the Client Satisfaction Questionnaire; UQ = the Internet Intervention Utility Questionnaire;

^b^ – = not report in the included study.

^c^ No adverse events were observed.
