# Supplementary material for: eHealth-Based Psychosocial Interventions for Adults With Insomnia: Systematic Review and Meta-analysis of Randomized Controlled Trials
Source: J Med Internet Res. 2023 Mar 14;25:e39250. doi: 10.2196/39250 (PMC10131777; doi:10.2196/39250)
Supplement: Multimedia Appendix 3 [file jmir_v25i1e39250_app3.docx]

**Multimedia Appendix 3**


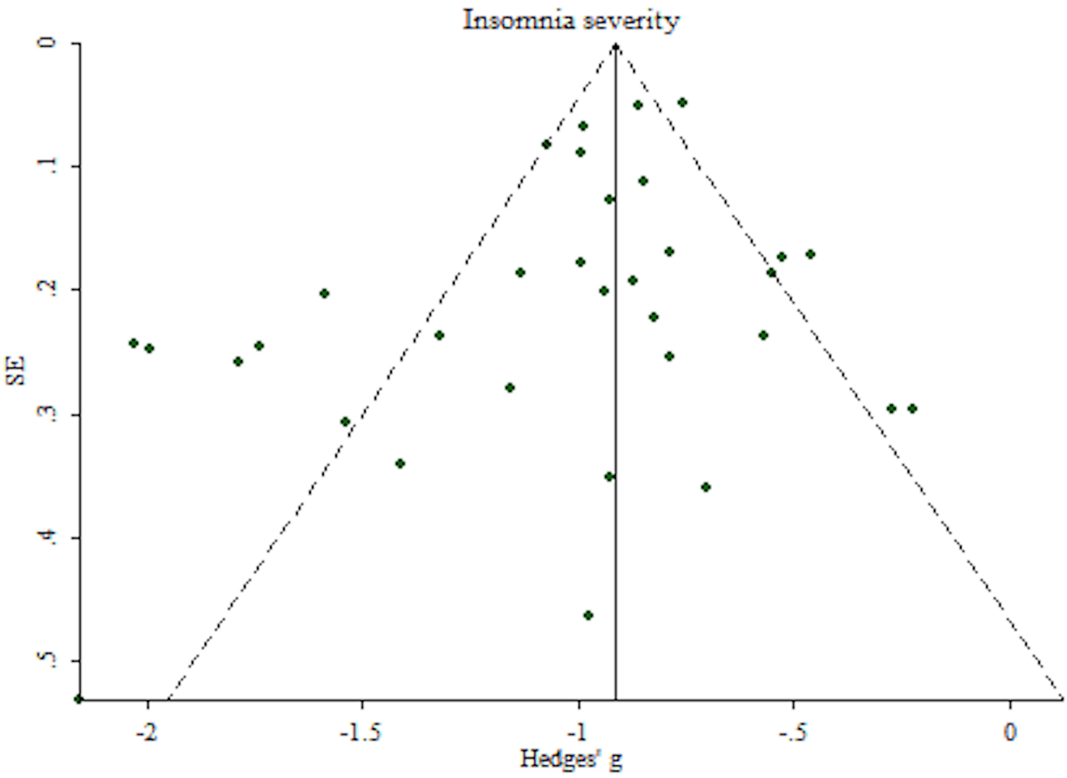


**
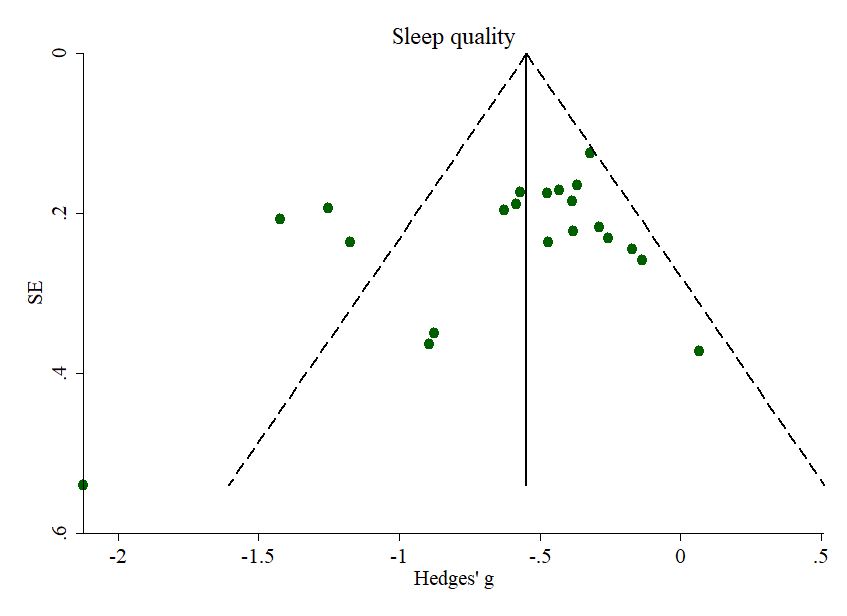
**

**Funnel plots of studies included in the meta-analyses.**

The vertical axis represents effect size and the horizontal axis means the standard error of effect size Horizontal line and sloping lines in funnel plot represent summary effect size and expected 95% CIs for a given standard error, respectively.
