# Supplementary material for: eHealth-Based Psychosocial Interventions for Adults With Insomnia: Systematic Review and Meta-analysis of Randomized Controlled Trials
Source: J Med Internet Res. 2023 Mar 14;25:e39250. doi: 10.2196/39250 (PMC10131777; doi:10.2196/39250)
Supplement: Multimedia Appendix 4 [file jmir_v25i1e39250_app4.docx]

**Multimedia Appendix 4**

**Insomnia severity**

**
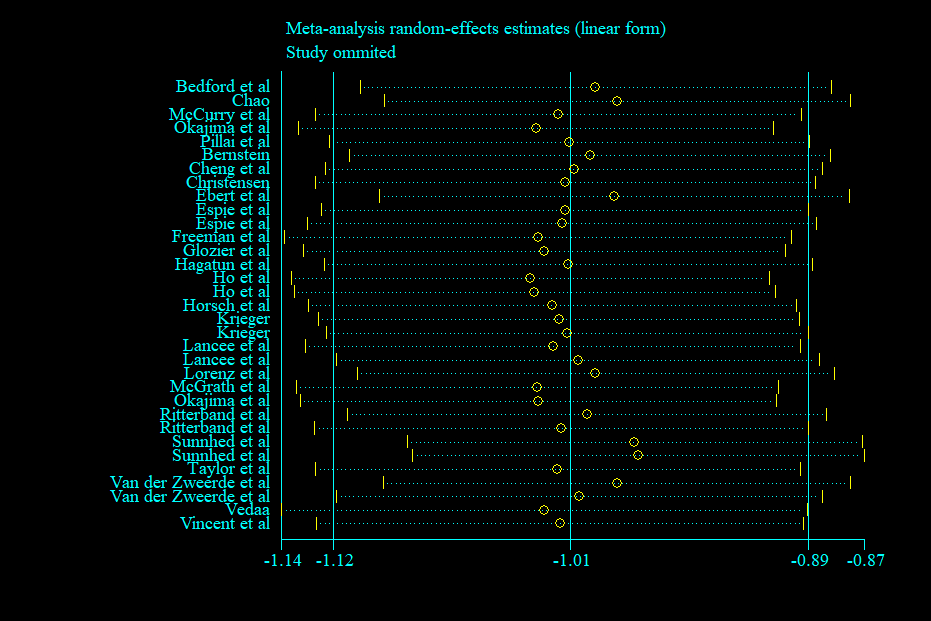
**

**Sleep quality**

**
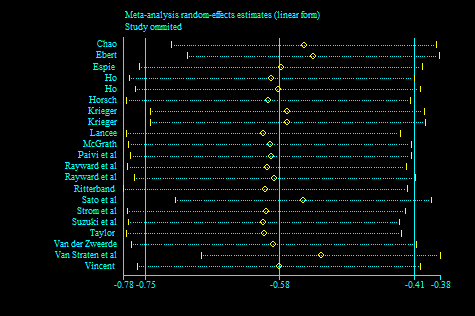
**

**Influence of individual studies on the effect size.**

The vertical axis indicates the overall effect size and the two vertical axes indicate its 95% CI. Every hollow round indicates the pooled effect size when the left study is omitted in this meta-analysis. The two ends of every broken line represent the respective 95% CI.
