# Supplementary material for: eHealth-Based Psychosocial Interventions for Adults With Insomnia: Systematic Review and Meta-analysis of Randomized Controlled Trials
Source: J Med Internet Res. 2023 Mar 14;25:e39250. doi: 10.2196/39250 (PMC10131777; doi:10.2196/39250)

**Multimedia Appendix 5**

**Plots of meta-regressions on insomnia severity**

**A Meta-regression of baseline severity.**


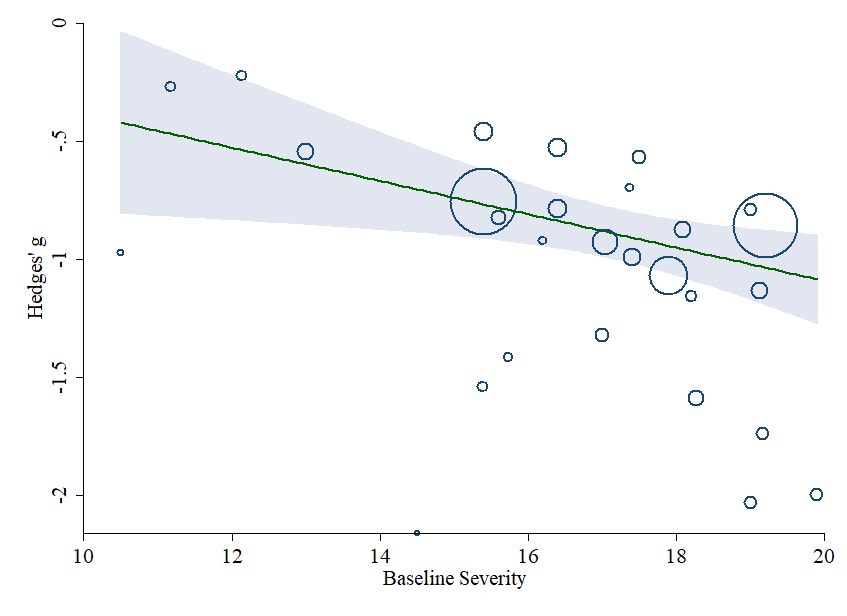


**B Meta-regression of intervention duration (in weeks).**


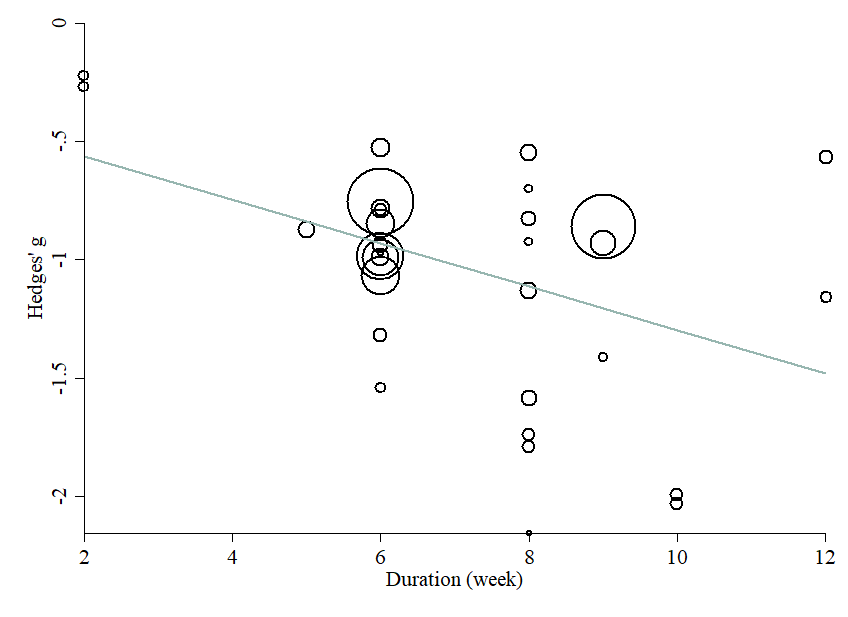


**C Meta-regression of intervention duration (in sessions).**


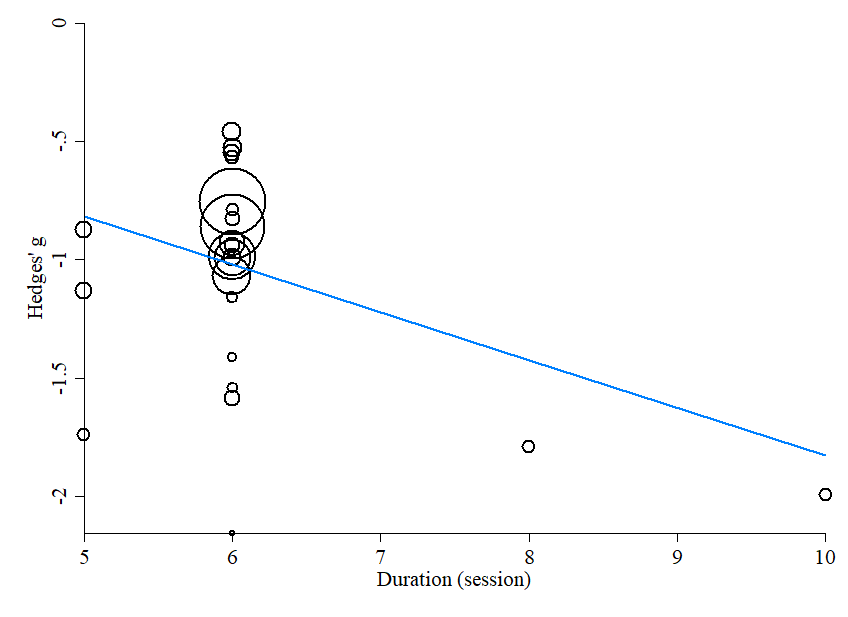

Supplement: Multimedia Appendix 5 [file jmir_v25i1e39250_app5.docx]
